# In Silico Optimization of Inhibitors of the 3-Chymotrypsin-like Protease of SARS-CoV-2

**DOI:** 10.3390/life16010006

**Published:** 2025-12-19

**Authors:** Issouf Fofana, Brice Dali, Mawa Koné, Katarina Sujova, Eugene Megnassan, Stanislav Miertus, Vladimir Frecer

**Affiliations:** 1 Laboratoire de Physique Fondamentale et Appliquée (LPFA), University of Abobo Adjamé (Now Nangui Abrogoua), 02 BP 801, Abidjan 02, Côte d’Ivoire; fofetude@yahoo.fr (I.F.);; 2 Laboratoire des Sciences de la Matière de l’Environnement et de l’énergie Solaire (LASMES), UFR SSMT Université Félix Houphouët Boigny, 22 BP 582, Abidjan 22, Côte d’Ivoire; 3 Laboratoire de Constitution et de Réaction de la Matière (LCRM), UFR SSMT Université Félix Houphouët Boigny, 22 BP 582, Abidjan 22, Côte d’Ivoire; 4 Department of Physical Chemistry of Drugs, Faculty of Pharmacy, Comenius University Bratislava, SK-83232 Bratislava, Slovakia; sujova23@uniba.sk; 5 International Centre for Applied Research and Sustainable Technology, SK-84104 Bratislava, Slovakia; 6 International Centre for Theoretical Physics, ICTP-UNESCO, Strada Costiera, I-34151 Trieste, Italy; 7 Department of Biotechnologies, Faculty of Natural Sciences, University of Ss. Cyril and Methodius, SK-91701 Trnava, Slovakia

**Keywords:** COVID-19, quantitative structure-activity relationship (QSAR), 3CL^pro^ protease inhibitors, pharmacophore (PH4), virtual screening of combinatorial library, ADME, molecular dynamics

## Abstract

In this study, new improved inhibitors of the viral enzyme 3-chymotrypsin-like protease (3CL^pro^) were designed using structure-based drug design techniques in an effort to discover more effective treatment of coronavirus disease 2019 (COVID-19). Three-dimensional models of 3CL^pro^–inhibitor complexes were prepared by in situ modification of the crystal structure of the submicromolar covalent inhibitor IPCL6 for a set of 25 known inhibitors with published inhibitory potencies (${IC}_{50}^{exp}$). The QSAR model was prepared with a reasonable correlation between the calculated free energies of formation of the 3CL^pro^-IPCL complex (∆∆*G*_com_) and the experimentally determined activities ${IC}_{50}^{exp}$, which explained approximately 92% of the variation in the 3CL^pro^ inhibition data. A similar agreement was achieved for the QSAR pharmacophore model (PH4) built on the basis of the active conformations of the IPCL inhibitors bound at the active site of the 3CL^pro^. The virtual combinatorial library of more than 567,000 IPCL analogues was screened in silico using the PH4 model and resulted in the identification of 39 promising analogues. The best inhibitors designed in this study show high predicted affinity for the 3CL^pro^ protease, as well as favourable predicted ADME properties. For the best new virtual inhibitor candidate IPCL 80-27-74-4, the inhibitory concentration ${IC}_{50}^{pre}$ was predicted equal to 0.8 nM, which represents a significant improvement in the inhibitory potency of known IPCLs. Ultimately, molecular dynamics simulations of the 12 newly designed top-scoring IPCL inhibitors demonstrated that the 3CL^pro^–inhibitor complexes exhibited good structural stability, confirming the potential for further development of the designed IPCL analogues.

## 1. Introduction

In this century, the world has experienced three major epidemics caused by coronaviruses: severe acute respiratory syndrome (SARS) in 2003, Middle East respiratory syndrome (MERS) in 2012, and the COVID-19 SARS-CoV-2 coronavirus pandemic in 2019 [1]. The SARS outbreak resulted in 8439 cases and 812 human deaths in 32 countries [2,3], MERS caused 2609 cases, including 939 deaths, while the global SARS-CoV-2 pandemic recorded more than 777.4 million cases and caused over 7 million deaths [4]. COVID-19 has had a significant global impact, affecting public health, healthcare systems, economies, and social structure around the world.

Successive emergence of several SARS-CoV-2 variants of concern, including the alpha, beta, gamma, delta, and omicron variants, in which mutations in the receptor binding domain of the viral spike protein (S) substantially increased its binding affinity to the human Angiotensin Converting Enzyme II (ACE2) receptor, has led to rapid spread of infection in the human population [5,6]. SARS-CoV-2 variants, such as the omicron, transmitted primarily through respiratory droplets and aerosols, have spread rapidly worldwide and are expected to evade the effectiveness of existing vaccines [5,6]. Other variants of interest include epsilon, zeta, eta, theta, iota, kappa, and lambda [7].

SARS-CoV-2 uses the S protein to bind to the ACE2 receptor present on the surface of human cells, primarily in the respiratory pathways [8,9]. This interaction allows the virus to enter the host cell, either through endocytosis or by direct fusion with the cell membrane [10,11]. Once inside the cell, the virus releases its genomic RNA into the cytoplasm [12]. This RNA is then used by the host cell machinery to replicate the viral genome and produce new viral particles [13,14]. The newly formed virions bud through the cell membrane, acquire a lipid envelope derived from the host cell, and are released to infect the neighbouring cells [15]. A key element in the viral replication process is the 3-chymotrypsin-like protease (3CL^pro^ main cysteine protease, a validated therapeutic target) [16]. This enzyme cleaves viral polyproteins into functional proteins necessary for viral RNA replication and transcription, making it a validated therapeutic target against COVID-19 [16]. It is highly specific to coronaviruses and is not present in humans [17]. SARS-CoV and SARS-CoV-2 3CL^pro^ are 96% identical at the amino acid level, including 100% identity within the catalytic pocket [18].

**Figure 1:**
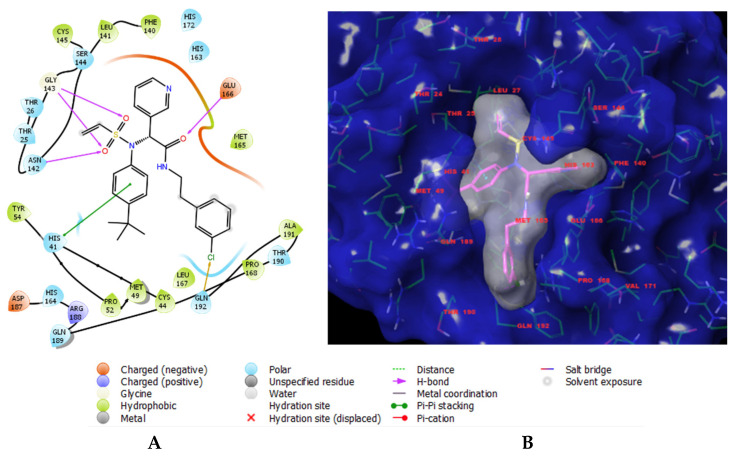
(**A**) Two-dimensional interaction scheme of inhibitor **14c** (IPCL1) [20] at the active site of the 3CL^pro^ protease of SARS-CoV-2. (**B**) Solid molecular surface of 3CL^pro^ (in blue), modelled in Insight-II [21], with the bound inhibitor IPCL1. The molecular surface of the IPCL1 inhibitor (in white) defines the occupied volume in the active site of 3CL^pro^. Carbon atoms are coloured magenta for the IPCL1 ligand and green for the protease 3CL^pro^. The side chains of the interacting residues and the ligand are represented as sticks.

Despite the significant progress made in the fight against COVID-19, the World Health Organisation considers that this disease remains a major health threat [4]. This underscores the urgency of intensifying research on more effective treatments against this virus and contributing to the UN Sustainable Development Goal 3 by combating communicable diseases by 2030 [19]. In this context, the present study aims to design new 3CL^pro^ protease inhibitors with enhanced efficacy and a favourable pharmacokinetic profile. To achieve this objective, we adopted a computer-aided molecular design (CAMD) approach for the evaluation of therapeutic molecules based on a training set of 20 known inhibitors (IPCLs) and a validation set of 5 inhibitors of 3CL^pro^ synthesised by Stille et al. [20]. First, we developed a QSAR model using the molecular mechanics Poisson–Boltzmann surface area (MM-PBSA) method, which uses the calculated Gibbs free energy of reversible enzyme–inhibitor complex formation as a single descriptor and the experimentally measured activities of IPCLs (${IC}_{50}^{exp}$). The most active compound in the series of SARS-CoV-2 3CL^pro^ protease inhibitors is compound **14c** (IPCL1), with an ${IC}_{50}^{exp}$ of 0.17 μM (Figure 1). Next, we generated a 3D-QSAR pharmacophore model (PH4) of 3CL^pro^ inhibition, based on the active conformation of the inhibitors within the active site. Subsequently, we developed a virtual combinatorial library of IPCL analogues and screened it in silico using the developed PH4 model to identify potent IPCL analogues. Finally, we probed the stability and flexibility of 3CL^pro^-IPCL complexes of the best analogues through molecular dynamics simulations.

## 2. Materials and Methods

**Scheme 1:**
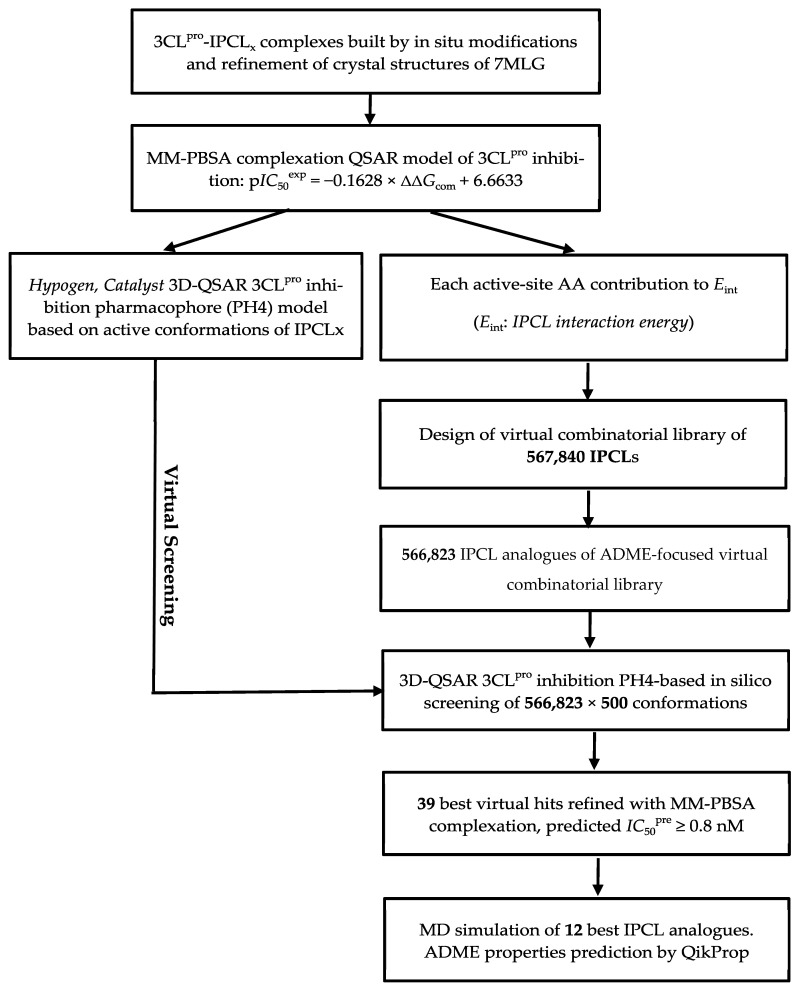
Flow diagram of the computer-aided drug design approach leading to the design of improved IPCL analogues that could potently inhibit the 3CL^pro^ of SARS-CoV-2.

The techniques and methods used in this study fall under computer-aided molecular design (CAMD) and optimisation of therapeutic molecules. They are based on the establishment of relationships between the structure and physicochemical properties of protein–ligand complexes and the known biological activities of a series of small-molecule inhibitors. This approach allows for the identification of substituents or structural modifications that may enhance relevant biological activity. Several studies available in the literature have successfully employed this methodology [22,23,24,25,26,27,28,29,30,31,32]. Scheme 1 summarises the key steps in the design of new and improved IPCL inhibitors.

### 2.1. Training and Validation Set of Inhibitors

The experimentally determined activities of the published IPCL inhibitors (${IC}_{50}^{exp}$) used in this work were obtained from a single study by Stille et al. [20]. The half-maximal inhibitory concentrations of the IPCLs cover a relatively wide range of three orders of magnitude (0.17 µM ≤ ${IC}_{50}^{exp}$ ≤ 45.1 µM) [20], allowing for the construction of a reliable QSAR model. To achieve this, 25 homologous IPCLs were divided into two sets using activity-based stratified sampling combined with structural diversity criteria, with 20 IPCLs assigned to the training set (TS) and five IPCLs to the validation set (VS), fulfilling the OECD criteria 80:20 ratio [33]. Both sets included active and inactive analogues.

### 2.2. Model Building

The refined crystal structure of the SARS-CoV-2 main protease 3CL^pro^ co-crystallised with the covalent inhibitor **14a** (IPCL6), obtained from the Protein Data Bank (PDB) under the PDB code 7MLG [34] (resolution 2.50 Å), was used to model the enzyme–inhibitor complexes (E:I*) included in the TS and VS by in situ modifications [32].

In the 3CL^pro^-IPCL6 complex (Figure S1 Supplementary Data), the electrophilic vinyl sulfonamide group of the inhibitor (**14a**) was covalently linked to the catalytic residue Cys145 [20,34]. This bond was removed since we have considered the attraction of a ligand binding to residues of the 3CL^pro^ active site rather than the ability of the IPCLs to covalently link to the catalytic Cys145. The Insight-II molecular modelling software [21] and Discovery Studio [35] were used in this work. The details of the preparation of the structures and the procedure for their optimisation, leading to stable noncovalent 3CL^pro^-IPCL associates, have been outlined in previous studies [28,32,36,37,38,39,40]. The Gibbs free energy of formation (GFE) of a reversible ligand–receptor complex E:I* is obtained according to Equation (1).

(1) $${∆∆G}_{com}={∆G}_{com}\left(I\right)-{∆G}_{com}\left(I_{ref}\right)={∆∆H}_{MM}-{∆∆TS}_{vib}+{∆∆G}_{sol}$$

The ∆∆*H*_MM_ describes the relative enthalpic contribution to change in GFE corresponding to the intermolecular interactions in the E:I* complex estimated by molecular mechanics (MM). The ∆∆*G*_sol_ and ∆∆*TS*_vib_ represent the relative solvation and vibrational entropy contributions to the GFE of the formation of the E:I* complex, respectively.

### 2.3. Molecular Mechanics

Molecular Mechanics (MM) was used for the modelling of the molecular structures of enzyme (E), inhibitors (I), and enzyme–inhibitor complexes (E:I*) as previously reported [26,28,31,32]. The class II consistent force field (CFFII) [41] was used to simulate the molecular structure and interatomic interactions with an all-atom representation. Polak–Ribière–Polyak conjugate gradient minimization method without any constraints and with strict converge criteria, dielectric constant of vacuum, and implicit GB/SA solvation model were used for the molecular geometry optimisation.

### 2.4. Conformational Search

The conformations of free inhibitors were obtained by relaxing the bound conformations in the E:I* complexes to a local energy minimum. A Monte Carlo search for low-energy conformations over all rotatable bonds, excluding the rings, generated 200 unique conformations. The conformer with the lowest energy was optimised through energy minimisation with a dielectric constant of 4. Subsequently, another optimisation of the conformer was performed with a dielectric constant of 80, taking into account solvent effects. The details of the conformational search have been previously reported [23,26,28,31].

### 2.5. Solvation Gibbs Free Energies

The electrostatic component of the solvation free energy (GFE) was calculated using the DelPhi module of Discovery Studio [35], taking into account the effect of ionic strength via the nonlinear Poisson–Boltzmann equation [42,43,44]. The solvent is modelled with a dielectric constant of 80, while the solute is enclosed within a molecular cavity characterised by a dielectric constant of 4. Calculations on cubic grids achieved a final resolution of approximately 0.3 Å per grid unit, using an ionic strength of a physiological medium of 0.145 mol.dm^−3^. The GFE was calculated as the reaction field energy [27,28,32,39,42,43,45]. The details have been reported previously [25,26,28,30,32].

### 2.6. Calculation of the Entropic Term

The entropic contribution during the binding of the inhibitor to the active site of the enzyme is calculated using a simplified method inspired by Fischer et al. [46,47], by analysing the normal modes of vibration of the inhibitor, as previously reported [26,28,48,49]. This calculation relies on the assessment of configurational entropy, which represents the number of accessible microstates, using statistical mechanics principles and considering normal modes of the ligands in vacuo and ligands constrained in the active site of the 3CL^pro^.

### 2.7. Calculation of Binding Affinity and QSAR Model

The formation of a reversible enzyme–inhibitor non-covalent adduct E:I* preceding the covalent attachment of IPCL to 3CL^pro^ (pre-equilibrium irreversible model of inhibitory effect E + I ↔ E:I* → EI [20]) is considered here to predetermine the inhibitory potencies of IPCL analogues, assuming comparable reactivity and kinetics of the electrophilic warheads towards the catalytic Cys145. The inhibitor shape, size, charge distribution, and initial position of the warhead appeared to be more important for the inhibitory effect than the warhead reactivity [20]. Thus, ${IC}_{50}^{pre}$ value of new IPCL analogues can be predicted using the calculated standard Gibbs free energy of reversible E:I* complex formation, assuming an equilibrium in aqueous solution. Details of the calculation of binding affinity and corresponding QSAR models have been previously reported [26,28,29,30,32,50].

### 2.8. Interaction Energy

The interaction energy calculation protocol (*E*_int_) that uses molecular mechanics analyses non-bonded interactions between the atoms of the enzyme and the atoms of the inhibitor in the reversible E:I* complexes. Pairwise interatomic interactions were evaluated using the CFFII force field [41] and the relative permittivity of 4 in Discovery Studio [35]. The decomposition of enzyme–inhibitor interaction energy into individual contributions from active site residues facilitates the identification of residues that influence the binding of ligands and helps to identify structural modifications of the IPCLs beneficial for the biological activity of the compounds [22].

### 2.9. Generation of Pharmacophore

A pharmacophore is made up of a set of essential functional groups arranged optimally, ensuring drug binding to its receptor and inducing a specific biological response [51,52]. Theoretically, drugs that bind to a particular receptor should share the same pharmacophore [53]. The evolution of the pharmacophore concept in the context of computer-aided drug design has been extensively documented [54,55,56]. Several approaches can be considered to identify a pharmacophore. In this study, we constructed a structure-based pharmacophore model, particularly the pharmacophore model associated with quantitative structure–activity relationship (QSAR). The bound conformations of inhibitors, derived from the E:I* complexes, were utilised to construct a pharmacophore using the HypoGen algorithm of Catalyst [57], integrated within Discovery Studio [35]. The HypoGen algorithm is commonly used to generate pharmacophore hypotheses based on binding models between inhibitors and their biological targets [26,35,36,37].

### 2.10. ADME Properties

The prediction of the pharmacokinetic profiles of new IPCL analogues was conducted using QikProp (Release 139) software by Schrödinger [58]. This programme was specifically designed to predict the pharmacokinetic properties of drug candidates utilising various physicochemical descriptors. The methodology employed relies on statistical models based on experimental data from more than 700 compounds, approximately 500 of which are established drugs along with related heterocycles [59,60,61,62,63]. The methods used for calculating pharmacokinetic properties in QikProp are based on predictive modelling techniques that incorporate regression algorithms to establish correlations between the physicochemical descriptors and the observed pharmacokinetic properties. According to Jorgensen [62,63], this approach has proven effective in accurately predicting the pharmacokinetic profiles and drug-likeness of emerging drug candidates. The chosen ADME descriptors were derived from the three-dimensional structures of the considered IPCLs.

### 2.11. Virtual Combinatorial Library Generation

The enumeration of the virtual combinatorial library was carried out using the appropriate module of Discovery Studio [35]. The R-groups were taken from the catalogue of building blocks in the CombiChem module of Discovery Studio [35]. Their tautomeric forms and protonation stated were adjusted to neutral pH. The R-groups were selected according to the properties of the targeted pockets S_4_-S_3_, S_2_, S_1_, and S_1_′ of the active site of 3CL^pro^ [34]. These molecular fragments were attached to the IPCL scaffold. The details of the construction of the virtual library have been previously reported [25,28,30,36,37].

### 2.12. Inhibitory Potency Prediction

IPCL conformers from the virtual combinatorial library that matched the PH4 pharmacophore were selected for in silico screening using the complexation QSAR model. The relative GFE of complexation in aqueous medium (∆∆*G*_com_) was calculated for the E:I* complex of each selected perspective analogue and used to estimate the inhibitory potencies (${IC}_{50}^{pre}$) employing target-specific scoring function parameterised for the 3CL^pro^ [25,29,36,37]:

(2) $${pIC}_{50}^{pre}=-{log}_{10}{IC}_{50}^{pre}=a\times ∆∆G_{com}+b$$

### 2.13. Molecular Dynamics Simulations

Molecular dynamics (MD) simulations serve as an essential tool in computational biology and chemistry, enabling researchers to examine the dynamic behaviour and stability of biomolecular complexes over time. Desmond from Schrödinger Inc. (New York, NY, USA) [64,65], is among the premier software applications for conducting high-performance molecular dynamics simulations. These simulations offer valuable insights into the structural and dynamic properties of molecular systems. Desmond is particularly noted for its speed and efficiency, especially when simulating large biomolecular systems [65]. Molecular dynamics was performed according to the protocols described previously [37,66,67].

## 3. Results

### 3.1. QSAR MODEL of 3CL^pro^ Inhibition

A set of 25 published IPCL micromolar 3CL^pro^ inhibitors, synthesised and tested for inhibitory potency against the 3CL^pro^ of SARS-CoV-2 by Stille et al. [20], were used to prepare a theoretical QSAR model of 3CL^pro^ inhibition. Experimentally measured ${IC}_{50}^{exp}$ values [20] were correlated with calculated relative GFE of formation of the 3CL^pro^-IPCL_x_ reversible complexes in aqueous solution (∆∆*G*_com_). The complete set of 25 IPCLs were divided into a training set (IPCL_1–20_) and a validation set of inhibitors (IPCL_21-25_). The inhibitory potency data span a relatively wide range (0.17 μM ≤ ${IC}_{50}^{exp}$ ≤ 45.1 μM), which is acceptable for the construction of a QSAR model. The composition of the considered inhibitors is presented in Table 1.

**Figure 2:**
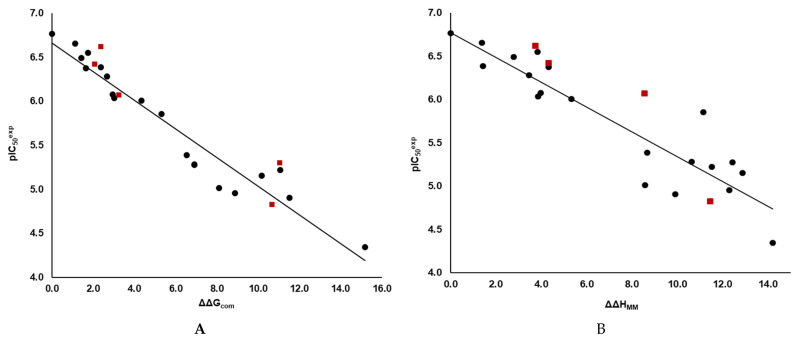
(**A**) Graph of the correlation between ${pIC}_{50}^{exp}$ and relative GFE of reversible E:I* complex formation ∆∆*G*_com_ for the 3CL^pro^-IPCL_x_ complexes [20]. (**B**) Similar correlation established for the enthalpic component ΔΔ*H*_MM_ of the relative GFE both in kcal·mol^−1^. The black points represent the data points from the training set, while the red squares indicate the data points from the validation set.

The calculated values of the GFE of formation of the 3CL^pro^-IPCL_x_ complexes and its contributions are presented in Table 2. These values were calculated according to the protocol described in the Materials and Methods section. Relative quantities were considered for each of the 25 inhibitors [20], taken with respect to the reference ligand IPCL1 (Table 1) to partially compensate for the approximate nature of the methods used [32]. The obtained QSAR models correlate experimentally measured inhibitory potencies of the IPCLs $p{IC}_{50}^{exp}=-{log}_{10}{IC}_{50}^{exp}$ [20] with the calculated relative GFE (∆∆*G*_com_) as well as its enthalpic component (∆∆*H*_MM_) derived from molecular models of enzyme–inhibitor complexes, Figure 2. The corresponding regression equations and their statistical evaluation of the correlations are given in Table 3 and indicate strong correlations between the calculated quantities (∆∆*H*_MM_, ∆∆*G*_com_) and the experimental observations $p{IC}_{50}^{exp}$. The established single-descriptor QSAR models show that the molecular models of the 3CL^pro^-IPCL_x_ complexes include the most important features, which are essential for the inhibitory potencies of IPCLs. The high coefficients of determination and Fisher test values for equations (A) and (B) in Table 3 suggest that the binding models explain the substantial portion of the variation in the inhibitory activities of IPCLs. The QSAR model [$p{IC}_{50}^{exp}$ = f(∆∆*G*_com_)] considers, besides ligand–receptor interactions (∆∆*H*_MM_), solvent effect (∆∆*G*_sol_) and vibrational entropic contribution to enzyme–inhibitor binding (∆∆*TS*_vib_) at T = 300 K. Incorporating entropy variation into QSAR models enhances the robustness of the models [48,68] and contributes to the robustness of predictions [49,69,70].

The quality of the developed binding model was verified by calculating the ratio of predicted to experimental activities ($p{IC}_{50}^{pre}/p{IC}_{50}^{exp})$ for the validation set of IPCLs, where ${pIC}_{50}^{pre}$ was calculated using regression equation B, Table 3. This ratio is close to the theoretical value of 1 for all IPCLs in the validation set (Table 2), corroborating the considerable predictive power of the complexation QSAR model. Thus, the correlation equation B and the calculated values of ∆∆*G*_com_ can be used to predict inhibitory potencies of new IPCL analogues, provided they share the same binding mode within the active site of 3CL^pro^ and share a similar chemical structure with the IPCL molecules from the training and validation sets. Moreover, the significant correlation obtained in this QSAR relationship has facilitated the determination of the active conformations of the IPCLs within the active site of 3CL^pro^ and has also enabled the generation of realistic pharmacophore model (PH4).

### 3.2. Binding Mode of IPCLs

**Figure 3:**
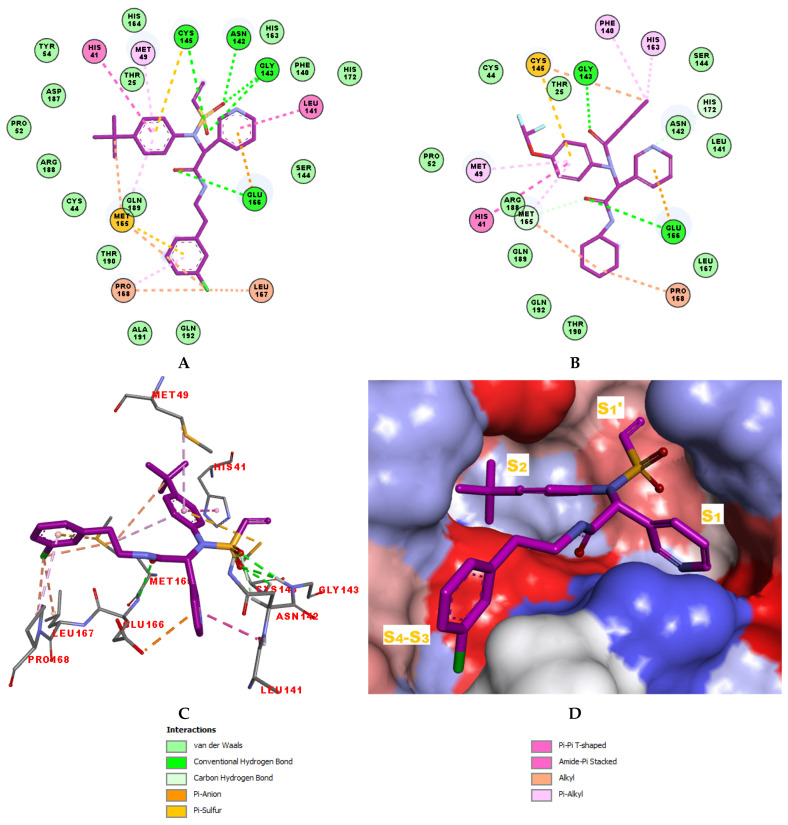
(**A**) Two-dimensional interaction diagram of the most potent inhibitor IPCL1 at the active site of 3CL^pro^. (**B**) Two-dimensional interaction diagram of the inhibitor IPCL20 at the active site of 3CL^pro^. (**C**) Three-dimensional structure of the 3CL^pro^ active site with bound reference inhibitor IPCL1. (**D**) Connolly surface of the 3CL^pro^ active site with the reference inhibitor IPCL1. Surface colouring legend: red—hydrophobic, blue—hydrophilic, white—intermediate. Carbon atoms are coloured magenta for the ligands IPCL1 and IPCL20, and grey for the side chains of the interacting residues of the 3CL^pro^ protease.

The structural information on enzyme–inhibitor interactions was obtained from the crystal structure of the 3CL^pro^-IPCL6 complex [34]. As illustrated in Figure 3D, in the S_1_′ pocket of the catalytic site, the inhibitor IPCL1 forms multiple hydrogen bonds between its vinylsulphonyl group and the catalytic residue Cys145, as well as residues Asn142 and Gly143 (Figure 3A,C). In contrast, the inhibitor IPCL20 in the S_1_′ pocket forms a hydrogen bond only with Gly143 through its 2-butynal group (Figure 3B). The 2-butynal group of IPCL20 also interacts via alkyl–alkyl interactions with the catalytic residue Cys145, as well as through π-alkyl interactions with residues Phe140 and His163 (Figure 3B). In the S_2_ pocket (Figure 3A), IPCL1 established multiple interactions with the active site residues. Notably, a π–π stacking interaction and a π–sulphur interaction occur between the phenyl group of the inhibitors and the catalytic residues His41 and Cys145 (Figure 3A–C) [71]. Additionally, two π–alkyl interactions were formed between the phenyl group of the inhibitors and residues Met49 and Met165 (Figure 3A–C). Similar interactions with 3CL^pro^ active site can be observed for most IPCLs, thanks to the presence of the common 4-*tert*-butylphenyl group in virtually all IPCLs considered. However, in the case of IPCL20, the *tert*-butyl group is replaced by a difluoromethoxy group. The resulting structural difference led to a loss of an alkyl–alkyl interaction between IPCL20 and Met165 (Figure 3B). In the S_1_ pocket, both IPCL1 and IPCL20 form a π–anion interaction between their pyridine group and Glu166 (Figure 3A–C). However, IPCL1 also forms a π–π stacking interaction between its pyridine group and residue Leu141 (Figure 3A–C), which is not present in the case of IPCL20. In the S_4_-S_3_ pocket, the cyclohexyl fragment of IPCL20 forms two alkyl-alkyl interactions with residues Met165 and Pro168 (Figure 3B), while the 2-(3-chlorophenyl)ethyl group of IPCL1 forms multiple interactions: a π–sulphur interaction with Met165, a π–alkyl interaction with Pro168, as well as three alkyl–alkyl interactions with residues Met165, Leu167, and Pro168 (Figure 3A–C). The molecular scaffold of IPCLs [20] forms a hydrogen bond with residue Glu166.

### 3.3. Interaction Energy

The calculated molecular mechanics inhibitor–residue interaction energy (Δ*E*_int_) between bound IPCL inhibitors and residues of the 3CL^pro^ active site for the training set measures the contributions of the enthalpic component of GFE to the overall enzyme–inhibitor binding free energy (Figure S2) [36,37,38,39]. Qualitative analysis of the Δ*E*_int_ contributions of IPCL_1-20_ revealed that some of the R-groups R3-R1′ of IPCLs occupying the pockets S_4_-S_3_-S_1_′ show elevated binding energy with the residues present in the corresponding pockets of the active site of 3CL^pro^. It also indicated that the largest portion of Δ*E*_int_ derives from the interaction of IPCLs with residues Glu166 (S_1_ pocket), Asn142 (S_1_′) and Gly143 (S_1_′). Additionally, dividing the interaction energy Δ*E*_int_ into van der Waals (Figure S3) and the remaining electrostatic contributions shows the character of the leading attractive interactions. The inhibitor–residue interaction energy is also useful for selecting suitable R-groups for each of the four binding pockets of the protease active site. This comparative analysis alone cannot suggest appropriate substitutions capable of enhancing the binding affinity; therefore, we developed and screened a virtual combinatorial chemical library of IPCL analogues using the established PH4 pharmacophore model [26,36,37,38].

### 3.4. Pharmacophore Model

The parameters of hypotheses of PH4 pharmacophore model of 3CL^pro^ protease inhibition generated using Discovery Studio [35] are shown in Table 4. These models were developed based on the active conformation of training set 20 inhibitors (IPCL_1-20_) and evaluated with the help of five validation set inhibitors (IPCL_21-25_). The process of generating the PH4 pharmacophore is detailed in the reference [37,38].

The Catalyst HypoGen algorithm [57] generated top 10 hypotheses, whose reliability was evaluated based on calculated cost parameters, ranging from 82.10 (Hypo1) to 224.63 (Hypo10). The CatScramble algorithm implemented in the Catalyst module of Discovery Studio [35] was used to validate the generated hypotheses. This process involved performing 49 random runs of each model, altering the positions of atoms and functional groups, and then assessing the quality of each model.

**Figure 4:**
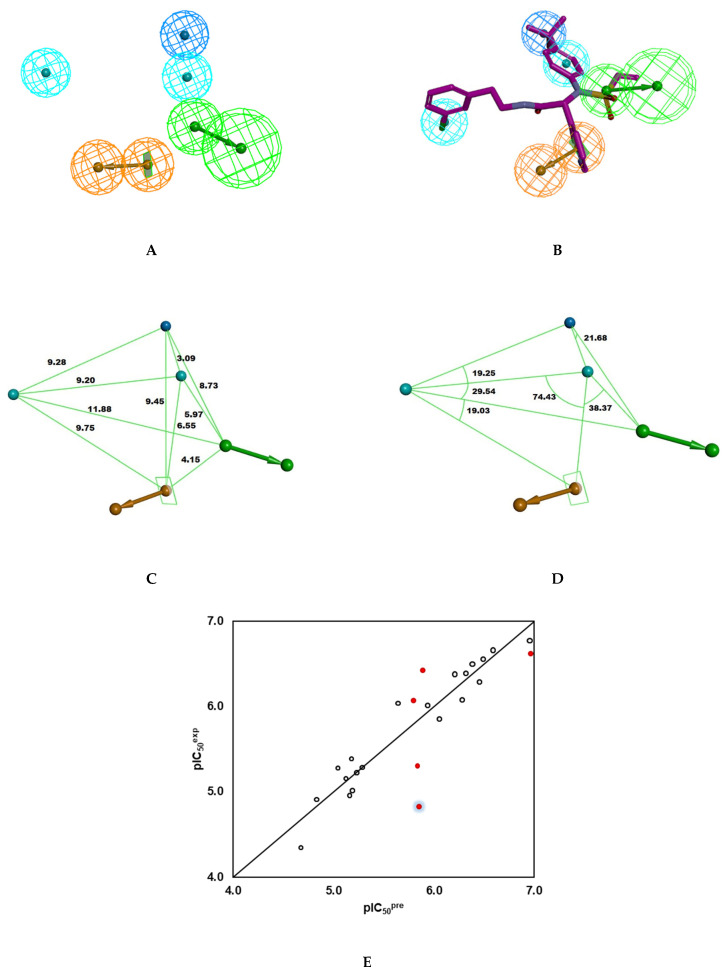
(**A**) Pharmacophore features, their centres, and vectors; HYD = hydrophobic (cyan), Ar = ring aromatic (orange), HBA = hydrogen bond acceptor (green), HYD-Al = Aliphatic hydrophobic (blue). (**B**) Mapping of the pharmacophore of 3CL^pro^ inhibitor and the most potent inhibitor IPCL1 [20]. Carbon atoms of the ligand IPCL1 are coloured magenta. (**C**) Distances between the centres of pharmacophoric features in [Å]. (**D**) Angles between the centres. (**E**) Correlation plot of experimental vs. predicted inhibitory activity using PH4 pharmacophore model (open circles correspond to Training Set, red dots to Validation Set).

The evaluation (Table 4) indicated that the generated pharmacophore models are significant with a confidence level of 98%, which implies that the identified pharmacophore features correspond with the observed inhibitory potencies ${IC}_{50}^{exp}$ of 20 IPCLs included in the training set. The number of scrambled runs (Y) required to satisfy the high confidence is defined by the relation S = [1 – (1 + X)/(1 + Y)] × 100%, where X is the number of hypotheses with a total cost below that of hypothesis 1 (Hypo1), which was set to zero (X = 0). Consequently, the total number of HypoGen runs (initial one + random runs) should equal 1 + Y = 50. The first hypothesis (Hypo1) of PH4, with a cost (170.21) closest to the fixed cost (45.55) exhibiting the best values of RMSD (1.855) and R^2^ = 0.97, was selected for further analysis. The resulting regression equation expresses ${pIC}_{50}^{exp}$ as a function of ${pIC}_{50}^{pre}$ estimated by Hypo1: ${pIC}_{50}^{exp}$ = 0.995 × ${pIC}_{50}^{pre}$ + 0.0285 (*n* = 20; R^2^ = 0.93; R_xv_^2^ = 0.92; *F*-test = 246.138; σ = 0.189, α > 98%), Figure 4E. Validation of the PH4 model with IPCL_21-25_ confirmed its predictive power, similar to that of the previously developed complexation QSAR model. Indeed, the calculated ratio (${pIC}_{50}^{pre}/{pIC}_{50}^{exp}$) for IPCL_21_ (1.05), IPCL_22_ (0.92), IPCL_23_ (0.95), IPCL_24_ (1.10), and IPCL_21_ (1.21) is close to theoretical value of 1, except for the last one (1.21) illustrated by the blue shading around the lowest red dot in Figure 4E. The Hypo1 which represents the 3CL^pro^ inhibition pharmacophore displays perfect superposition of the active centres over the 3D structure of the most active inhibitor from the training set IPCL1, Figure 4B.

The complexation QSAR model and the 3CL^pro^ inhibition pharmacophore model provide insights into the structural requirements governing the inhibitory potencies of IPCLs. Thus, the PH4 pharmacophore of 3CL^pro^ inhibition can be used to screen the virtual combinatorial library of IPCL analogues in order to identify new, more potent IPCLs. The predicted potencies of the most perspective new potential inhibitors will be estimated using the linear regression Equation (B), Table 3, of the complexation QSAR model from computed enzyme–inhibitor binding affinities ∆∆*G*_com_.

### 3.5. Virtual Combinatorial Library of IPCLs and In Silico Screening

An initial virtual combinatorial library (VCL) was generated by substituting function groups at the R_1_′, R_1_, R_2_, and R_3_ positions (Table 1) of the IPCL scaffold with new R-groups listed in Table 5. The R-groups were carefully chosen considering hydrophobicity, shape, size and amino acid composition of each pocket of the 3CL^pro^ active site [34]. The process resulted in a VCL of the size R_1_′ × R_1_ × R_2_ × R_3_ = 24 × 26 × 26 × 35 = 567,840 analogues. Generated IPCL analogues resemble the training set of IPCL molecules in terms of composition, size, and chemical structure (see Table 2 and Table 3 in Ref. [20]). The initial VCL underwent a refinement by applying Lipinski’s rule of five [72], with a molecular weight threshold set at *M*_w_ ≤ 725 g·mol^−1^. The reduced VCL that contained 566,823 analogues was then subjected to in silico screening in Discovery Studio [35] with the established PH4 (Hypo1) model to identify new analogues that match the pharmacophore model well. The in silico pharmacophore-based screening of a VCL was demonstrated to lead to the identification of virtual hits, as demonstrated by our previous studies on inhibitor design [23,24,27,31,39]. During in silico screening, 500 conformers were generated for each element of the VCL. After the screening, 1576 IPCLs were mapped to at least three pharmacophoric features of the PH4 Hypo1 pharmacophore model of the 3CL^pro^ inhibition (Figure 4A,B). Among them, the top 39 analogues (PH4 hits) were retained and subjected to screening using the QSAR complexation model. The GFE of complex formation ∆∆*G*_com_ with the 3CL^pro^ was calculated and predicted inhibitory concentrations ${IC}_{50}^{pre}$ were estimated using regression Equation (B), Table 3. Calculated ∆∆*G*_com_, their contributions, and the resulting predicted inhibitory potencies of the virtual hits are given in Table 6.

### 3.6. New IPCL Analogues

**Figure 5:**
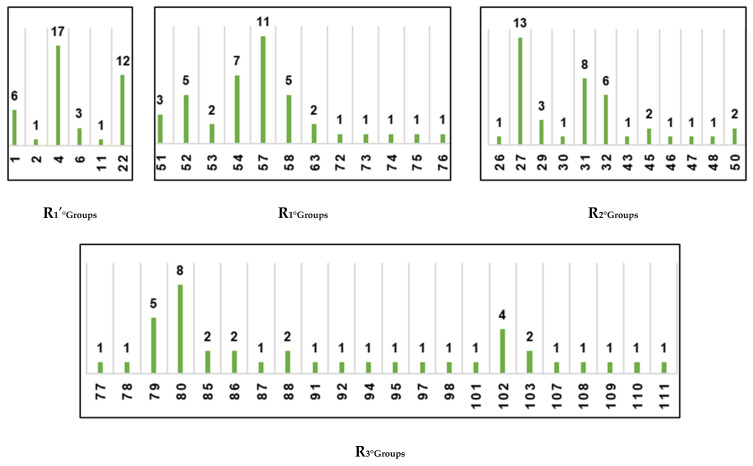
Histograms of frequency of occurrence of individual R-groups in the 39 best selected analogues mapping to features of the PH4 pharmacophore hypothesis Hypo1 (for the structures of the fragments see Table 5).

To identify R-groups (substituents) leading to virtual hits (new potent inhibitor candidates), we prepared histograms representing the frequency of occurrence of groups R_3_-R_1_′ among the top 39 PH4 matches (Figure 5). Analysis of the histograms revealed that the most frequent fragments at the R_1_′ position were 1, 4, and 22. At the R_2_ position, the predominant fragments were 27, 31, and 32. The R_1_ position of virtual hits was mostly occupied by fragments 57, 54, 52, and 58. Finally, for position R_3_, the most suitable fragments were 79, 80, and 102.

An analysis of the structural requirements for inhibiting 3CL^pro^ showed that the substituents at the R_3_ position of most potent known inhibitors [20] did not make sufficient use of hydrophobic contacts available in the explored S_4_-S_3_ pocket of the active site (Figure 3D). Therefore, the new IPCL analogues that align with the inhibition pharmacophore of 3CL^pro^ and better fill the occupied S_4_-S_3_ pocket could serve as more potent inhibitors (Table 6). The substituents of R_1_′, R_1_, and R_2_ positions of the best designed analogues also demonstrate elevated affinity to the respective residues of the S_1_′, S_1_, and S_2_ pockets of the active site of SARS-CoV-2 3CL^pro^.

**Figure 6:**
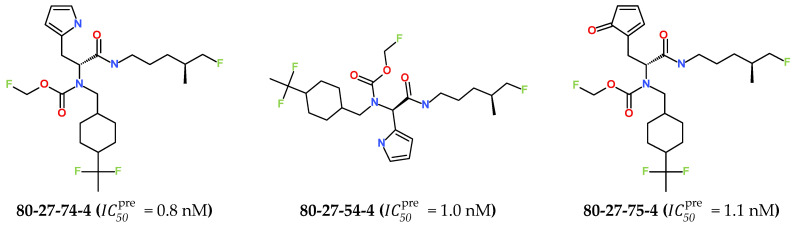

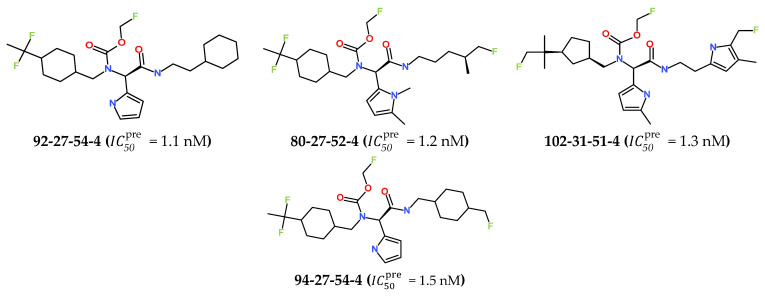
Chemical structures of the seven best inhibitors designed.

The best virtually designed IPCL analogues (Table 6, Figure 6) include the following: **80-27-74-4** (${IC}_{50}^{pre}$ = 0.8 nM), 80-27-54-4 (${IC}_{50}^{pre}$ = 1.0 nM), **80-27-75-4** (${IC}_{50}^{pre}$ = 1.1 nM), **92-27-54-4** (${IC}_{50}^{pre}$ = 1.1 nM), **80-27-52-4** (${IC}_{50}^{pre}$ = 1.2 nM), **102-31-51-4** (${IC}_{50}^{pre}$ = 1.3 nM), and **94-27-54-4** (${IC}_{50}^{pre}$ = 1.5 nM). The best designed analogue, **80-27-74-4** exhibits a predicted inhibitory potency approximately 200 times higher than the best compound of the training set, IPCL1 (${IC}_{50}^{exp}$ = 170 nM). A reason for this large increase in the predicted potency of the **80-27-74-4** was examined by analysing interactions of this analogue with the residues of the active site of 3CL^pro^.

**Figure 7:**
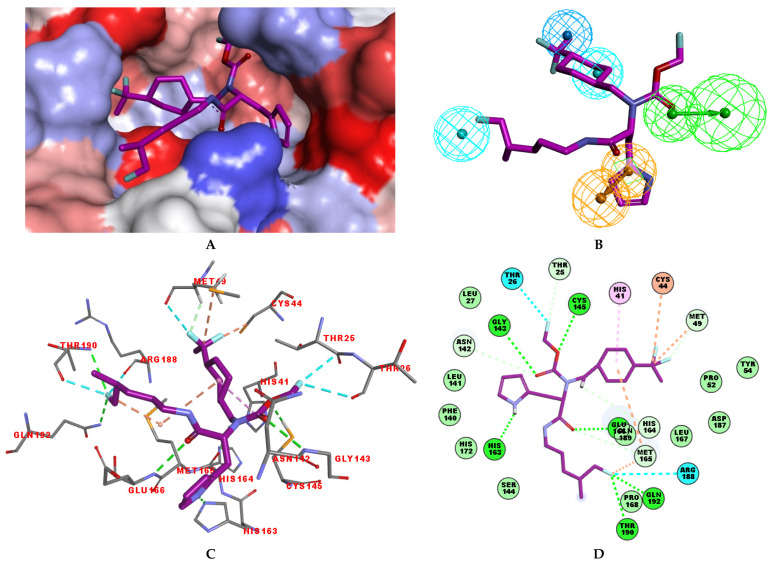
(**A**) Connolly surface of the active site of SARS-CoV-2 3CL^pro^ with bound most active designed IPCL analogue **80-27-74-4** (${IC}_{50}^{pre}$ = 0.8 nM). The molecular surface of the binding site is coloured according to residue hydrophobicity: red—hydrophobic, blue—hydrophilic, and white—intermediate. (**B**) Mapping of the IPCL **80-27-74-4** to the 3CL^pro^ inhibition pharmacophore. (**C**) Close up and interactions of the virtual hit IPCL **80-27-74-4** at the active site of 3CL^pro^. (**D**) Two-dimensional schematic interaction diagram of the IPCL **80-27-74-4** at the active site of 3CL^pro^. Carbon atoms are coloured magenta for the ligand IPCL **80-27-74-4**.

In the S_1_′ pocket of the 3CL^pro^ active site, the substituent (**4**) fluoromethoxycarbonyl (Table 5) at position R_1_′ establishes two hydrogen bonds with residue Gly143 and the catalytic residue Cys145, Figure 7D. It also forms two halogen bonds with residues Thr25 and Thr26. These interactions contribute to stabilising fragment **4** within the S_1_′ pocket of the 3CL^pro^ active site. In the S_2_ pocket, the substituent (**27**) [4-(1,1-difluoroethyl)cyclohexyl]methyl at position R_2_ interacts with several residues (Figure 7C,D). A halogen bond is formed between a fluorine atom and residue Met49. Furthermore, a hydrophobic π-alkyl interaction occurs between the cyclohexyl of substituent (**27**) and the catalytic residue His41. There are also three alkyl-type hydrophobic interactions with residues Cys44, Met49 and Met165. In the S_1_ pocket of the 3CL^pro^ active site, the substituent (**74**) 1H-pyrrol-2-ylmethyl at position R_1_ interacts with residue His163 via a hydrogen bond. In the S_4_-S_3_ pocket, the substituent (**80**) [(4S)-5-fluoro-4-methylpentyl] at position R_3_ establishes several stabilising interactions with the residues of this pocket (Figure 7C,D). Residues Thr190 and Gln192 interact with the fluorine atom of this substituent via two hydrogen bonds. Residues Arg188 and Thr190 form halogen-hydrophobic contacts with the fluorine atom of substituent (**80**), while an alkyl-type hydrophobic contact is observed between this fluorine atom and residue Met165. The Glu166 residue forms a hydrogen bond with the molecular scaffold of the IPCLs (Figure 7C,D). These numerous hydrophobic contacts and hydrogen bonds explain the elevated affinity of IPCL **80-27-74-4** with the active site residues, significantly contributing to its stability within 3CL^pro^.

**Figure 8:**
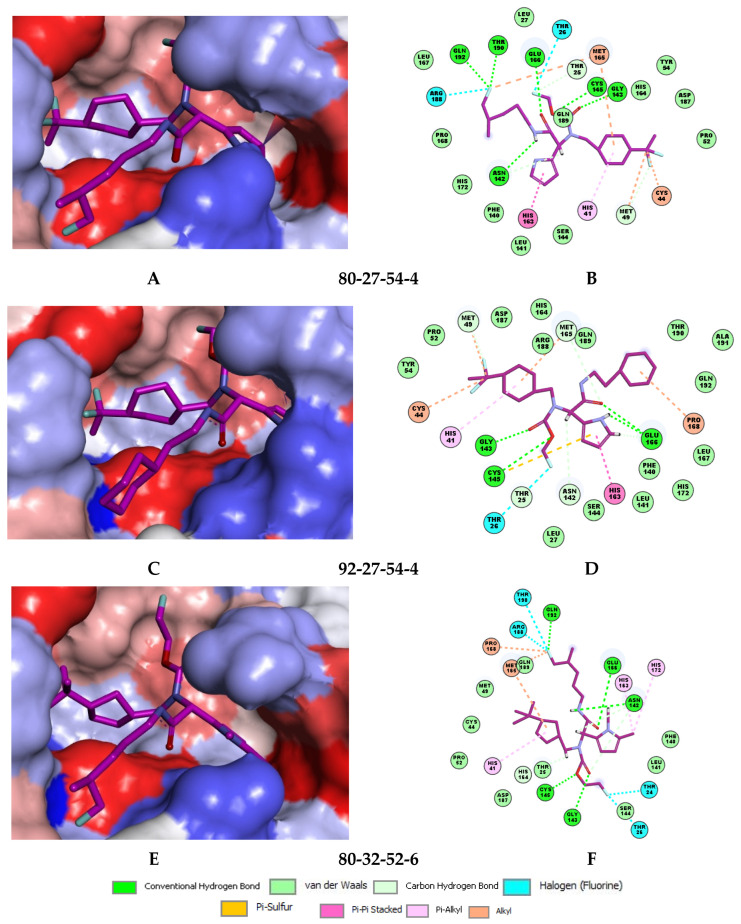
(**A**) Connolly surface of the active site of SARS-CoV-2 3CLpro with one of the best designed IPCL analogues: **80-27-54-4** (${\mathrm{IC}}_{50}^{\mathrm{pre}}$ = 1.0 nM). (**B**) Two-dimensional interaction diagram of **80-27-54-4** at the active site of 3CLpro. (**C**) Connolly surface of the 3CLpro active site with one of the best-designed IPCL analogues: **92-27-54-4** (${\mathrm{IC}}_{50}^{\mathrm{pre}}$ = 1.1 nM). (**D**) Two-dimensional interaction diagram of **92-27-54-4** at the active site of 3CLpro. (**E**) Connolly surface of the 3CLpro active site with one of the best-designed IPCL analogues: **80-32-52-6** (${\mathrm{IC}}_{50}^{\mathrm{pre}}$ = 2.2 nM). (**F**) Two-dimensional interaction diagram of **80-32-52-6** at the active site of 3CLpro. The binding site surface is colour-coded according to the hydrophobicity of the residues: red—hydrophobic, blue—hydrophilic, and white—intermediate. Carbon atoms are coloured magenta for the three ligand IPCLs.

Several stabilising interactions are observed between the substituents of other three promising IPCL analogues and the residues of the active site of SARS-CoV-2 3CL^pro^ (Figure 8). Among them, a π–π stacking interaction is found between the substituent (**54**) 1H-pyrrol-2-yl at position R_1_ of IPCLs **80-27-54-4** and **92-27-54-4**, and the residue His163. Additionally, a π–sulphur interaction is formed between the 1H-pyrrol-2-yl and the catalytic Cys145. Attractive interactions are also observed between the substituent (**6**) 2-fluoroethoxycarbonyl at position R_1_′ of IPCL **80-32-52-6** and the residues of the S_1_′ pocket (Figure 8). Indeed, the 2-fluoroethoxycarbonyl substituent establishes two hydrogen bonds with the residue Gly143 and the catalytic residue Cys145. Furthermore, it forms two halogen bonds between the fluorine atom of **6** and the residues Thr24 and Thr26. A hydrogen bond interaction formed between the Glu166 residue and the molecular scaffold carbonyl of the IPCLs is observed in all studied 3CL^pro^-IPCL_x_ complexes (Figure 8).

Attractive interactions between the substituents of the best designed IPCL analogues and the residues of the 3CL^pro^ active site are also highlighted by the interaction energy diagram, which shows the individual Δ*E*_int_ contributions (Figure S4). By comparing interaction energy diagrams of the IPCL1 (the best inhibitor in the training set) and the residues of the active site, with the best designed new IPCL analogues, we can conclude that the new IPCLs exhibit increased electrostatic interactions with most of the active site residues.

### 3.7. Predicted Pharmacokinetic Profile of New IPCL Analogues

Evaluation of the ADME properties of drug candidates at an early stage of a drug discovery project is essential for successful drug development, as they determine the rates of drug absorption, distribution in tissues, metabolic conversion, and excretion. We have calculated a set of 24 ADME-related descriptors using QikProp software [58], following the method of Jorgensen [59,60,61,62,63], for the newly designed IPCL analogues, as well as for IPCL1 (the best inhibitor in the training set) and for drugs used for the treatment of COVID-19 or currently undergoing clinical trials. In Table 7, we present 16 of the 24 calculated descriptors for our 12 best designed IPCL analogues, and the reference drugs. The low values of the overall drug-likeness descriptor (#*stars*) indicate that the new IPCL analogues exhibit acceptable predicted ADME-related properties. Among these descriptors, the percentage of human oral absorption in the gastrointestinal tract (%*HOA*), which defines the oral bioavailability of drugs, is more favourable for the new IPCL analogues compared to drugs used for COVID-19 treatment or compounds under clinical investigation. Therefore, we can recommend the synthesis and biological evaluation of the best designed IPCL analogues with a probable drug-like character.

### 3.8. Molecular Dynamics Simulations

**Figure 9:**
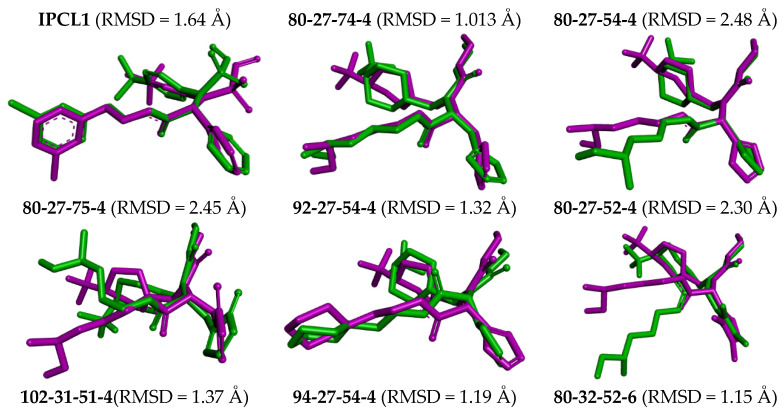

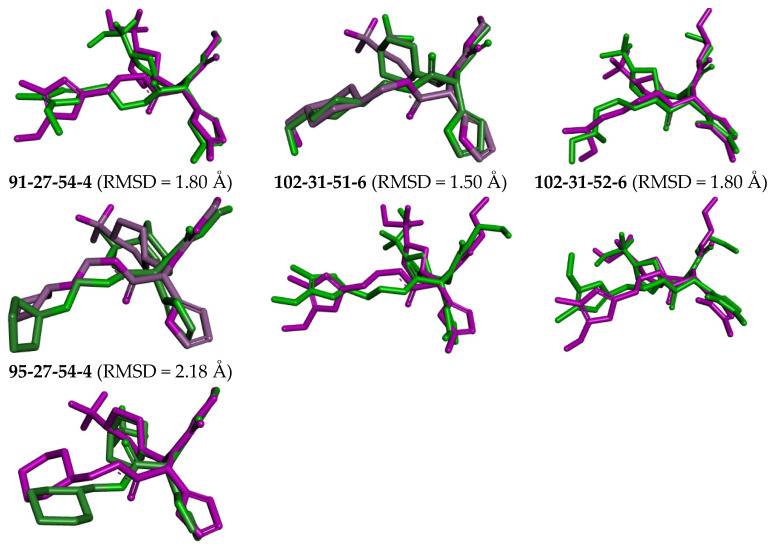
Overlay of the averaged bound conformation of selected new IPCLs from MD simulation (green colour) and those derived from the crystal structure of 3CL^pro^-IPCL complex 7MLG (magenta).

The complexes of the 3CL^pro^ with bound inhibitors IPCL1 [20] and IPCLs 80-27-74-4, 80-27-54-4, 80-27-75-4, 92-27-54-4, 80-27-52-4, 102-31-51-4, 94-27-54-4, 80-32-52-6, 91-27-54-4, 102-31-51-6, 102-31-52-6, and 95-27-54-4 (Table S2) were analysed via 200 ns molecular dynamics simulations using Desmond programme [66] to assess stability of bound conformations of the inhibitors considered. Initial geometries were prepared by in situ modification of the crystal structure of 3CL^pro^-IPCL6 complex [20,34] and refined using molecular mechanics. Subsequently, the MD simulations enabled calculation of dynamic parameters by analysing the MD trajectory (total/potential energy, RMSD, radius of gyration, evolution of protein–ligand interactions, Figures S5–S7) [65,66,67]. The analysis revealed stability of bound IPCL inhibitors (maximum value of RMSD < 2.4 Å, Figure S5) with the fluctuations attributable to thermal motions. The time evolution of the enzyme–inhibitor complexes highlighted the stabilising interactions of the residues Glu166, Asn142, and Gly143 with IPCLs, which formed hydrogen bonds and water bridges and thus can contribute to the inhibitor specificity for 3CL^pro^ (Figures S6 and S7). Superposition of post-simulation averaged inhibitor conformations (Figure 9) showed only minor structural deviations (RMSD < 2.5 Å), suggesting the stability of the modelled complexes and validity of the molecular model of action in vitro. These findings support the potential of IPCLs as inhibitors of the viral protease SARS-CoV-2 3CL^pro^.

## 4. Discussion

The analysis of the binding mode of the best designed potential inhibitors identified several interactions that might be important for the 3CL^pro^ inhibition. Our study identified new IPCL inhibitors with higher affinity for residues in the active site of 3CL^pro^. The increased affinity of the best-designed inhibitors is illustrated by the 2D interaction mapping of enzyme–inhibitor interactions (Figure 7 and Figure 8) and confirmed by the interaction energy diagram (Figure S4). Indeed, the diagram suggests that the residues His41, Ser144, and His163 form stronger electrostatic interactions with some of the designed IPCL analogues than with the reference IPCL1. Our approach enabled the identification of suitable molecular substituents occupying the pockets of the 3CL^pro^ active site. The substituents (**4**) fluoromethoxycarbonyl and (**6**) 2-fluoroethoxycarbonyl (Table 5) interact favourably with the residues in the S_1_′ pocket (Figure 7 and Figure 8), particularly with the catalytic residue Cys145, which plays a crucial role in the inhibition of 3CL^pro^. This observation was confirmed by Aly et al. [73]. Furthermore, substituents **4** and **6** form a carbamate group with the nitrogen atom of the scaffold, which displays electrophilic character and is potentially capable of forming bonds with nucleophiles, such as the catalytic residue Cys145 in the S_1_′ pocket [20,66,74,75,76,77,78]. In the S_1_ pocket, substituents (**54**) 1H-pyrrol-2-yl and (**74**) 1H-pyrrol-2-ylmethyl exhibit attractive interactions with the residues Glu166 and His163 (Figure 7 and Figure 8). It is interesting to note that the residue Glu166, interacting via a hydrogen bond with all the best designed new IPCL inhibitors, plays a crucial role in the inhibition of the 3CL^pro^ protease [74,79,80]. Furthermore, it participates in substrate recognition and in binding with inhibitors [20,80,81,82]. The interaction of Glu166 with the R_1_ group was observed in at least 20% of the 500 frames analysed from the MD simulations (Figure S7) for the best designed potential inhibitors. Sencanski et al. [83] emphasise that this interaction is essential for effectively orienting the ligand in the S_1_ pocket of the substrate binding site. The substituent (**27**) [4-(1,1-difluoroethyl)cyclohexyl]methyl demonstrated a strong affinity for the residues in the S_2_ pocket of the active site, due to the stabilising π–alkyl type interactions and halogen bonds formed with the catalytic residue His41 and residues Met49, Cys44, and Met165 (Figure 7 and Figure 6). According to our previous study [66], the establishment of hydrogen bonds between the flanking substituent (**80**) [(4S)-5-fluoro-4-methyl-pentyl] and the S_4_-S_3_ pocket residues Thr190 and Gln192 (Figure 7 and Figure 8) contributes to the stability of the 3CL^pro^–inhibitor complexes. Liu et al. [84] state that the hydrogen bond between the residue Asn142 and the ligands (Figure 8) is important for the binding of inhibitors at the 3CL^pro^ active site.

Predicted inhibitory potency of the best designed IPCL 80-27-74-4 against the 3CL^pro^ of SARS-CoV-2 ${IC}_{50}^{pre}$ = 0.8 nM is comparable to the experimental enzymatic ${IC}_{50}$ of Nirmatrelvir reported in the literature to range between 2 and 15 nM, depending on the assay conditions [85]. Our computational prediction of inhibitory potencies is based on calculated relative Gibbs free energy of receptor binding (∆∆*G*_com_) and the QSAR model of 3CL^pro^ inhibition (Table 3). Since the complexation QSAR model was trained on IPCLs with a restricted range of experimental potencies (${IC}_{50}^{exp}$ of 0.17–45.1 μM) and the calculated ∆∆*G*_com_ is in part molecular size-dependent quantity, the predicted low nanomolar affinities of the best IPCL analogues may be overestimated compared to experimental wet-lab results.

## 5. Conclusions

The need for developing COVID-19 treatments remains important due to the continued emergence of new coronavirus variants and their lasting impact on the vulnerable part of the population. This study identified new, improved IPCL inhibitors of the 3CL^pro^ of SARS-CoV-2, with predicted half maximal inhibitory concentrations in the low nanomolar range. The identification of these new molecules was achieved by combining several computational methods of rational drug design. Using the crystal structure of the 3CL^pro^-IPCL6 complex [20,34], a QSAR model of 3CL^pro^ inhibition was constructed, which correlated the calculated Gibbs free energies of reversible enzyme–inhibitor complex formation with experimentally measured inhibitory potencies [20]. A PH4 pharmacophore model was developed from the bound conformations of 20 IPCLs included the training set [20]. The analysis of the geometric characteristics and properties of each of the four binding pockets of the 3CL^pro^ active site enabled the identification of suitable substituents (R-groups) that were used to create a virtual library of over 567,000 new IPCL analogues. Screening of the virtual library using the pharmacophore model led to the identification of 39 top-scoring new IPCL analogues. Their inhibitory potencies were predicted based on the calculated relative Gibbs free energies ∆∆*G*_com_.

This strategy resulted in the identification of new substituents occupying four pockets of the 3CL^pro^ active site, including (**27**) [4-(1,1- difluoroethyl)cyclohexyl]methyl (pocket S_2_), (**4**) fluoromethoxycarbonyl and (**6**) 2-fluoroethoxycarbonyl (pocket S_1_′), (**54**) 1H-pyrrol-2-yl and (**74**) 1H-pyrrol-2-ylmethyl (pocket S_1_), and (**80**) [(4S)-5-fluoro-4-methyl-pentyl] (pocket S_3_). These substituents demonstrated high affinity for the residues of 3CL^pro^ active site, as supported by numerous hydrogen bonds and hydrophobic contacts observed between the inhibitor molecules and enzyme residues in the complex structures.

Molecular dynamics simulations support the stability of the 3CL^pro^-IPCL complexes formed by the best designed new analogues and thus reinforce the ability of the new analogues to occupy the catalytic site of 3CL^pro^ and to suppress its normal function. The new IPCLs also exhibit favourable predicted ADME-related properties and show probable drug-like character. Low inhibition of the ubiquitous human cysteine protease cathepsin L by IPCLs of the training set [20] confirmed excellent selectivity against 3CL^pro^ over cathepsin L. We can therefore assume that some level of selectivity for 3CL^pro^ is maintained in the structurally similar inhibitors designed in this work. We therefore recommend that medicinal chemistry laboratories proceed with their synthesis and inhibitory potency testing to evaluate their potential use as new COVID-19 treatments.

## Figures and Tables

**Table 1:** Training set (TS) and validation set (VS) of IPCL inhibitors [20] used for the development of the QSAR model of 3CL^pro^ inhibition. IPCL molecular scaffold and building blocks (R-groups R_1_′, R_1_, R_2_, and R_3_) are shown.

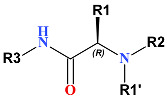							
**No.**	**1**	**2**	**3**	**4**	**5**	**6**	**7**
**R-Group**	** 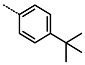 **	** 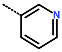 **	** 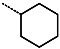 **	** 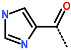 **	** 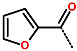 **	** 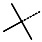 **	** 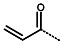 **
**No.**	**8**	**9**	**10**	**11**	**12**	**13**	
**R-Group**	** 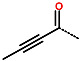 **	** 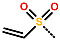 **	** 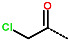 **	** 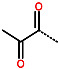 **	** 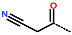 **	** 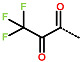 **	
**No.**	**14**	**15**	**16**	**17**	**18**	**19**	
**R-Group**	** 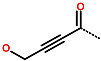 **	** 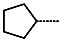 **	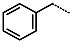	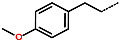	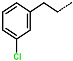	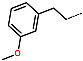	
**No.**	**20**	**21**	**22**	**23**	**24**	**25**	**26**
**R-Group**	** 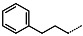 **	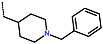	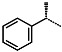	** 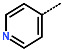 **	** 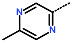 **	** 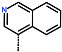 **	** 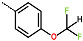 **
**Training set**	**IPCL1**	**IPCL2**	**IPCL3**	**IPCL4**	**IPCL5**	**IPCL6**	**IPCL7**
**R_3_–R_2_–R_1_–R_1_′**	18-1-2-9	21-1-2-9	17-1-2-9	22-1-2-9	3-1-2-10	3-1-2-9	20-1-2-9
${IC}_{50}^{exp}$ (µM)	0.17	0.22	0.28	0.32	0.41	0.42	0.52
**Training set**	**IPCL8**	**IPCL9**	**IPCL10**	**IPCL11**	**IPCL12**	**IPCL13**	**IPCL14**
**R_3_–R_2_–R_1_–R_1_′**	3-1-23-10	16-1-2-10	3-1-24-10	6-1-2-5	3-1-2-4	3-1-2-11	3-1-2-8
${IC}_{50}^{exp}$ [µM]	0.84	0.92	0.98	1.40	4.1	5.2	5.3
**Training set**	IPCL15 *	**IPCL16**	**IPCL17**	**IPCL18**	**IPCL19**	**IPCL20**	
**R_3_–R_2_–R_1_–R_1_′**	22-1-2-9	3-1-2-12	15-1-2-8	3-1-2-7	3-1-2-13	3-26-2-8	
${IC}_{50}^{exp}$ [µM]	6.0	7.0	9.7	11.1	12.4	45.1	
**Validation set**	**IPCL21**	**IPCL22**	**IPCL23**	**IPCL24**	**IPCL25**		
**R_3_–R_2_–R_1_–R_1_′**	19-1-2-9	15-1-2-10	3-1-2-14	3-1-25-8	6-1-2-8		
${IC}_{50}^{exp}$ [µM]	0.24	0.38	0.85	5.0	15.0		

* In the case of IPCL15, the chiral centre at R1 has an (S) configuration.

**Table 2:** Complexation Gibbs free energy (binding affinity, **∆∆*****G*****_com_**) and its components calculated for the training set of SARS-CoV-2 3CL^pro^ inhibitors IPCL_1-20_ and validation set inhibitors IPCL_21-25_ [20].

**Training Set ^a^**	***M*** **_w_ ^b^**	**∆∆** ***H*** **_MM_ ^c^**	**∆∆** ***G*** **_sol_ ^d^**	**ΔΔ** ***TS*** **_vib_ ^e^**	**∆∆** ***G*** **_com_ ^f^**	${IC}_{50}^{exp}$ **^g^**
	**[g·mol^−1^]**	**[kcal·mol^−1^]**	**[kcal·mol^−1^]**	**[kcal·mol^−1^]**	**[kcal·mol^−1^]**	**[µM]**
IPCL1 *	512	0.0	0.0	0.0	0.0	0.17
IPCL2	547	1.4	1.5	1.7	1.1	0.22
IPCL3	508	3.8	1.3	3.4	1.8	0.28
IPCL4	478	2.8	1.0	2.4	1.4	0.32
IPCL5	442	1.4	2.7	1.7	2.4	0.41
IPCL6	456	4.3	0.7	3.4	1.7	0.42
IPCL7	492	3.5	3.1	3.9	2.7	0.52
IPCL8	437	4.0	−0.9	0.2	3.0	0.84
IPCL9	450	3.9	−0.9	0.0	3.0	0.92
IPCL10	452	5.3	−1.6	−0.6	4.3	0.98
IPCL11	434	11.1	−6.5	−0.7	5.3	1.4
IPCL12	460	8.7	−0.2	1.9	6.5	4.1
IPCL13	436	10.6	0.8	4.5	6.9	5.2
IPCL14	432	12.4	−2.1	3.4	6.9	5.3
IPCL15	479	11.5	−1.9	−1.4	11.1	6
IPCL16	433	12.9	0.7	3.5	10.2	7
IPCL17	418	8.6	2.0	2.4	8.1	9.7
IPCL18	420	12.3	−0.3	3.1	8.9	11.1
IPCL19	490	9.9	0.3	−1.3	11.5	12.4
IPCL20	441	14.2	−0.8	−1.8	15.2	45.1
**Validation Set**	***M*** **_w_ ^b^**	**∆∆** ***H*** **_MM_ ^c^**	**∆∆** ***G*** **_sol_ ^d^**	**ΔΔ** ***TS*** **_vib_ ^e^**	**∆∆** ***G*** **_com_ ^f^**	$p{IC}_{50}^{pre}/p{IC}_{50}^{exp}$ **^h^**
	**[g·mol^−1^]**	**[kcal·mol^−1^]**	**[kcal·mol^−1^]**	**[kcal·mol^−1^]**	**[kcal·mol^−1^]**	
IPCL21	508	3.7	2.6	4.0	2.4	0.95
IPCL22	428	4.3	−0.2	2.0	2.1	0.99
IPCL23	448	8.6	−1.2	4.1	3.3	1.01
IPCL24	482	15.3	0.0	4.3	11.1	0.91
IPCL25	406	11.5	2.1	2.8	10.7	1.02

(*) Reference compound IPCL1: ∆*H*_MM_^i^ = −51.8 kcal∙mol^−1^; ∆*G*_sol_^j^ = 9.0 kcal·mol^−1^; ∆*TS*_vib_ ^k^ = 1.7 kcal·mol^−1^; ∆*G*_com_^l^ = −44.5 kcal·mol^−1^. ^a^ for the chemical structures of the training and validation sets of inhibitors see Table 1; ^b^ *M*_w_ is the molecular mass of inhibitors; ^c^ ΔΔ*H*_MM_ is the relative enthalpic contribution to the GFE change related to reversible E:I* complex formation derived by MM: ΔΔ*H*_MM_ ⋍ [*E*_MM_{E:I_x_*} − *E*_MM_{I_x_}] − [*E*_MM_{E:I_ref_*} − *E*_MM_{I_ref_}], I_ref_ is the reference inhibitor IPCL1; ^d^ ΔΔ*G*_sol_ is the relative solvent effect contribution to the GFE change in reversible E:I* complex formation: ΔΔ*G*_sol_ ⋍ [*G*_sol_{E:I_x_*} − *G*_sol_{I_x_}] − [*G*_sol_{E:I_ref_*} − *G*_sol_{I_ref_}]; ^e^ −ΔΔ*TS*_vib_ is the relative entropic contribution of inhibitor I_x_ to the GFE of reversible E:I_x_* complex formation: ΔΔ*TS*_vib_ = [*TS*_vib_{I_x_}_E_ − *TS*_vib_{I_x_}] − [*TS*_vib_{I_ref_}_E_ − *TS*_vib_{I_ref_}]; ^f^ ΔΔ*G*_com_ is the overall relative GFE change in reversible E:I_x_* complex formation: ΔΔ*G*_com_ = ΔΔ*H*_MM_ + ΔΔ*G*_sol_ − ΔΔ*TS*_vib_; ^g^ ${IC}_{50}^{exp}$ is the experimentally measured half-maximal inhibitory concentration of IPCL obtained from Ref. [20]; ^h^ ratio of predicted and experimental half-maximal inhibition concentrations ${{pIC}_{50}^{pre}/pIC}_{50}^{exp}$ (${pIC}_{50}^{pre}=-\log_{10}{IC}_{50}^{pre}$) was predicted from computed ΔΔ*G*_com_ using the regression equation B for IPCL shown in Table 3; ^i^ Δ*H*_MM_ = *E*_MM_{E:I_x_*} − *E*_MM_{E} − *E*_MM_{I_x_}; ^j^ Δ*G*_sol_ = *G*_sol_{E:I_x*_} − *G*_sol_{E} − *G*_sol_{I_x_}; ^k^ Δ*TS*_vib_ = *TS*_vib_{I_x_}_E_ − *TS*_vib_{I_x_}; ^l^ Δ*G*_com_ = Δ*H*_MM_ + Δ*G*_sol_ − Δ*TS*_vib_.

**Table 3:** Regression analysis of computed binding affinities ∆∆*G*_com_, its enthalpic component ∆∆*H*_MM_ *, and experimental half-maximal inhibitory concentrations ${pIC}_{50}^{exp}=-\log_{10}{IC}_{50}^{exp}$ of IPCLs towards 3CL^pro^.

Statistical Data of Linear Regression	(A)	(B)
${pIC}_{50}^{exp}=-0.1430\times ∆∆H_{MM}+6.7722$ (A)		
${pIC}_{50}^{exp}=-0.1628\times ∆∆G_{com}+6.6633$ (B)		
Number of compounds *n*	20	20
Squared correlation coefficient of regression $R^{2}$	0.84	0.93
Leave-one-out cross-validated squared predictive correlation coefficient $Q_{xv}^{2}$	0.81	0.91
Standard error of regression *σ*	0.290	0.193
Statistical significance of regression, Fisher *F*-test	94.021	235.338
Level of statistical significance *α*	>95%	>95%
Range of half-maximal inhibitory concentrations ${IC}_{50}^{exp}$ [µM]	0.17–45.1	
Squared correlation coefficient of regression of validation set $R_{ext}^{2}$	0.75	0.90
Predictive squared correlation coefficient of regression of validation set $Q_{ext}^{2}$	0.53	0.85
Root mean square error of validation set ${RMSE}_{ext}$	0.47	0.26
Mean absolute error of validation set $M{AE}_{ext}$	0.44	0.21
**Aggregated metrics of QSAR model (B) for iterative reshuffling of the TS and vs. (Y-Randomization)**		
Squared correlation coefficient of regression $R^{2}$		0.92 ± 0.02
Leave-one-out cross-validated squared predictive correlation coefficient $Q_{xv}^{2}$		0.90 ± 0.02
Predictive squared correlation coefficient of regression of validation set $Q_{ext}^{2}$		0.80 ± 0.33
Root mean square error of validation set ${RMSE}_{ext}$		0.21 ± 0.06
Mean absolute error of validation set ${MAE}_{ext}$		0.18 ± 0.05

* ∆∆*G*_com_ and ∆∆*H*_MM_ are in [kcal·mol^−1^].

**Table 4:** Parameters of ten generated PH4 pharmacophoric hypotheses for the 3CL^pro^ inhibition after passing CatScramble validation procedure.

Hypothesis	RMSD ^a^	*R* ^2 b^	Total Costs ^c^	Costs Difference ^d^	Closest Random ^e^	Features ^f^
Hypo1	1.855	0.97	82.10	457.86	170.21	HBA-HYD_Al-HYD-HYD-RING_Ar
Hypo2	2.845	0.92	130.92	409.04	193.30	HBA-HYD_Al-HYD-HYD-RING_Ar
Hypo3	3.550	0.87	176.24	363.73	199.38	HBA-HYD_Al-HYD-HYD-RING_Ar
Hypo4	3.580	0.86	178.80	361.16	256.42	HBA-HYD_Al-HYD-HYD-RING_Ar
Hypo5	3.635	0.86	181.48	358.48	265.72	HBA-HYD_Al-HYD-HYD-RING_Ar
Hypo6	3.784	0.85	192.02	347.95	286.74	HBA-HYD_Al-HYD-HYD-RING_Ar
Hypo7	3.768	0.85	193.96	346.01	294.29	HBA-HYD_Al-HYD-HYD-RING_Ar
Hypo8	4.065	0.82	215.69	324.28	300.22	HBA-HYD_Al-HYD-HYD-RING_Ar
Hypo9	4.146	0.81	222.35	317.62	321.99	HBA-HYD_Al-HYD-HYD-RING_Ar
Hypo10	4.156	0.81	224.63	315.34	323.16	HBA-HYD_Al-HYD-HYD-RING_Ar
Fixed Cost	0	1	45.55	494.42		
Null Cost	6.991	0	539.97	0		

^a^ root mean square deviation; ^b^ squared correlation coefficient; ^c^ overall cost parameter of the PH4 pharmacophore; ^d^ cost difference between null cost and hypothesis total cost; ^e^ lowest cost from 49 scrambled runs at a selected level of confidence of 98%. The Fixed cost = 45.55 with RMSD = 0, the Null cost = 539.97 with RMSD = 7.103 and the Configuration cost = 9.036; ^f^ HBA (hydrogen-bond acceptor); HYD_Al (hydrophobic aliphatic); HYD (hydrophobic); RING_Ar (Ring_Aromatic).

**Table 5:** R_1_′-R_3_-groups (building blocks) used in the design of the initial diversity virtual combinatorial library of IPCL.

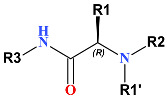						
**R-Group ^a,b^**						
**No.**	**1**	**2**	**3**	**4**	**5**	**6**
**R-Group**	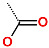	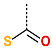	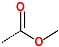	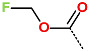	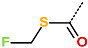	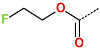
**No.**	**7**	**8**	**9**	**10**	**11**	**12**
**R-Group**	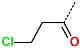	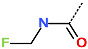	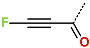	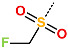	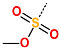	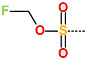
**No.**	**13**	**14**	**15**	**16**	**17**	**18**
**R-Group**	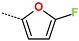	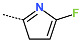	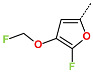	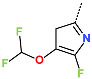	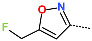	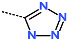
**No.**	**19**	**20**	**21**	**22**	**23**	**24**
**R-Group**	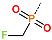	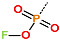	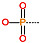	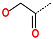	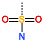	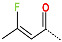
**No.**	**25**	**26**	**27**	**28**	**29**	**30**
**R-Group**	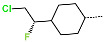	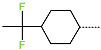	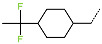	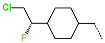	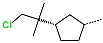	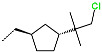
**No.**	**31**	**32**	**33**	**34**	**35**	**36**
**R-Group**	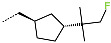	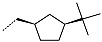	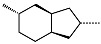	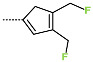	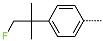	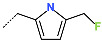
**No.**	**37**	**38**	**39**	**40**	**41**	**42**
**R-Group**	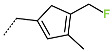	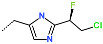	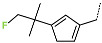	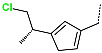	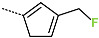	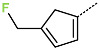
**No.**	**43**	**44**	**45**	**46**	**47**	**48**
**R-Group**	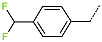	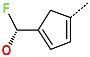	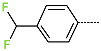	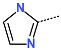	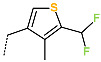	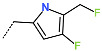
**No.**	**49**	**50**	**51**	**52**	**53**	**54**
**R-Group**	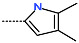	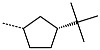	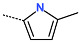	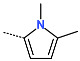	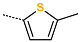	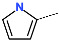
**No.**	**55**	**56**	**57**	**58**	**59**	**60**
**R-Group**	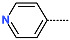	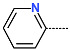	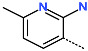	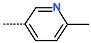	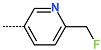	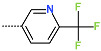
**No.**	**61**	**62**	**63**	**64**	**65**	**66**
**R-Group**	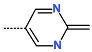	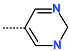	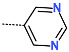	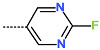	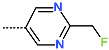	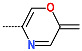
**No.**	**67**	**68**	**69**	**70**	**71**	**72**
**R-Group**	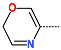	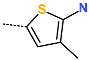	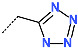	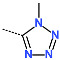	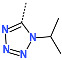	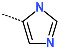
**No.**	**73**	**74**	**75**	**76**	**77**	**78**
**R-Group**	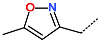	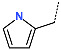	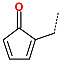	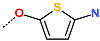	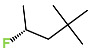	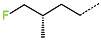
**No.**	**79**	**80**	**81**	**82**	**83**	**84**
**R-Group**	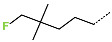	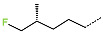	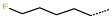	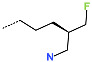	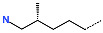	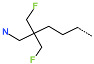
**No.**	**85**	**86**	**87**	**88**	**89**	**90**
**R-Group**	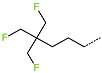	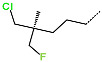	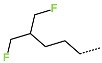	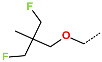	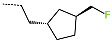	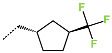
**No.**	**91**	**92**	**93**	**94**	**95**	**96**
**R-Group**	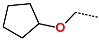	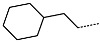	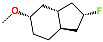	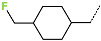	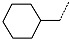	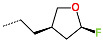
**No.**	**97**	**98**	**99**	**100**	**101**	**102**
**R-Group**	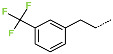	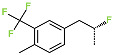	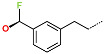	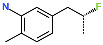	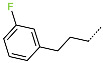	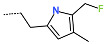
**No.**	**103**	**104**	**105**	**106**	**107**	**108**
**R-Group**	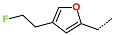	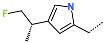	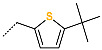	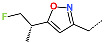	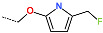	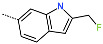
**No.**	**109**	**110**	**111**			
**R-Group**	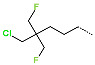	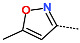	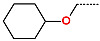			

^a^ Fragments 1 to 24 were utilised at R_1_′, fragments 25 to 50 at R_2_, fragments 51 to 76 at R_1_, and fragments 77 to 111 at R_3_. ^b^ The dashes indicate the points of attachment.

**Table 6:** Complexation GFE and their components for the top-scoring 39 IPCL virtual hits. The analogue numbering concatenates the index of each substituent R_3_ to R_1_′ with the substituent numbers taken from Table 5.

New IPCL Analogues	R_3_-R_2_-R_1_-R_1_′ R-Groups	*M*_w_ ^a^	∆∆*H*_MM_^b^	∆∆*G*_sol_^c^	∆∆*TS* ^d^	∆∆*G*_com_^e^	${IC}_{50}^{pre}$ ^f^
		[g·mol^−1^]	[kcal·mol^−1^]	[kcal·mol^−1^]	[kcal·mol^−1^]	[kcal·mol^−1^]	[nM]
Ref.	IPCL1	512	0	0	0	0	170 ^g^
1	79-32-57-22	493	−0.6	4.3	9.9	−6.3	20.3
2	85-32-57-22	529	−4.2	3.6	6.7	−7.3	13.5
3	85-30-57-22	563	−5.9	5.0	4.9	−5.8	23.7
4	87-32-57-22	511	−3.5	4.6	7.8	−6.7	17.1
5	79-31-57-22	511	−4.5	5.0	7.6	−7.2	14.4
6	86-32-57-22	527	−2.3	2.9	8.0	−7.4	13.0
7	86-31-57-22	545	2.2	2.1	6.2	−2.0	103.6
8	109-31-57-22	563	1.2	2.7	4.5	−0.5	177.4
9	88-50-57-22	499	3.2	1.0	10.1	−5.9	23.3
10	79-32-57-4	511	−0.2	0.7	9.5	−8.9	7.5
11	79-31-57-4	529	−2.7	1.1	6.8	−8.4	9.0
12	79-48-52-4	489	−6.5	−0.5	0.7	−7.7	11.9
13	111-47-73-22	500	−1.7	−4.4	−0.7	−5.4	28.3
14	80-27-53-22	491	−2.6	4.1	7.2	−5.7	25.0
15	80-27-53-4	509	−4.6	2.6	6.7	−8.7	8.0
16	80-27-52-22	488	−0.6	3.9	11.0	−7.6	12.0
**17**	**80-27-52-4**	**506**	**−6.7**	**−0.1**	**6.9**	**−13.7**	**1.2**
**18**	**80-27-75-4**	**505**	**−6.1**	**−0.3**	**7.7**	**−14.0**	**1.1**
**19**	**80-27-74-4**	**492**	**−13.1**	**1.6**	**3.1**	**−14.6**	**0.8**
20	77-50-76-4	490	9.9	4.2	7.0	7.1	3250.6
21	80-32-52-6	498	−1.0	−0.4	10.8	−12.1	2.2
22	78-26-58-11	494	4.5	−0.9	4.0	−0.3	192.2
23	102-31-52-6	553	−7.4	−0.3	3.1	−10.9	3.5
24	102-31-51-6	539	−18.3	10.1	3.0	−11.2	3.1
**25**	**102-31-51-4**	**525**	**−17.7**	**9.4**	**5.2**	**−13.4**	**1.3**
26	102-31-51-1	493	−3.7	5.1	9.1	−7.8	11.5
27	88-29-58-1	504	−1.1	−0.6	6.3	−8.0	10.4
28	103-29-58-4	526	2.7	0.9	3.9	−0.3	191.1
29	103-29-58-1	494	4.0	0.9	1.4	3.5	833.4
30	108-43-58-1	496	−1.9	−1.2	−7.1	3.9	967.9
31	98-45-63-1	526	4.5	−2.3	−6.5	8.8	6024.8
32	97-45-63-1	494	4.9	−2.8	−4.6	6.6	2653.6
**33**	**80-27-54-4**	**478**	**−9.6**	**2.1**	**6.6**	**−14.2**	**1.0**
34	91-27-54-4	474	-8.2	1.2	4.8	−11.8	2.4
**35**	**92-27-54-4**	**486**	**−5.4**	**−0.5**	**8.1**	**−14.0**	**1.1**
**36**	**94-27-54-4**	**504**	**−8.9**	**2.3**	**6.6**	**−13.2**	**1.5**
37	95-27-54-4	472	−4.0	0.9	6.6	−9.7	5.5
38	101-27-54-4	512	−1.2	1.6	2.4	−1.9	104.0
39	107-27-54-4	503	−7.0	−0.3	−2.7	−4.6	38.0

^a^ *M*_w_ is molecular mass; ^b^ ΔΔ*H*_MM_ is the relative enthalpic contribution to the GFE change in the 3CL^pro^-IPCL complex formation ΔΔ*G*_com_ (for details see footnote in Table 2); ^c^ ΔΔ*G*_sol_ is the relative solvation contribution to ΔΔ*G*_com_; ^d^ ΔΔ*TS*_vib_ is the relative entropic (vibrational) contribution to ΔΔ*G*_com_; ^e^ ΔΔ*G*_com_ is the relative Gibbs free energy change related to the enzyme- inhibitor 3CL^pro^-IPCL complex formation ΔΔ*G*_com_ ≅ ΔΔ*H*_MM_ + ΔΔ*G*_sol_ − ΔΔ*TS*_vib_. ^f^ ${IC}_{50}^{pre}$ is the predicted inhibitory potency towards 3CL^pro^ calculated from ΔΔ*G*_com_ using correlation equation B, Table 3; ^g^ ${IC}_{50}^{exp}$ [20] is given for the reference inhibitor IPCLs instead of the ${IC}_{50}^{pre}$.

**Table 7:** Predicted ADME-related properties of the best-designed IPCL analogues and known antiviral agents computed by QikProp [58]. See Table S1 for designed IPCLs.

IPCL_x_^a^	#*stars* ^b^	*M*_w_^c^ [g·mol^−1^]	*S*_mol_^d^ [Å^2^]	*S*_mol,hfo_^e^ [Å^2^]	*V*_mol_^f^ [Å^3^]	*RotB* ^g^	*HB*_don_ ^h^	*HB*_acc_ ^i^	log*P*_o/w_ ^j^	log*S*_wat_^k^	log*K*_HSA_^l^	log*B/B* ^m^	*BIP*_caco_ ^n^ [nm·s^−1^]	#*meta* ^o^	${\mathrm{IC}}_{50}^{\mathrm{pre}}$ [nM] ^p^	*HOA* ^q^	%*HOA* ^r^
IPCL1	1	512.1	848.6	98.3	1568.7	10	1	8.5	4.9	−6.7	0.4	−1.0	899.3	3	170 *	1	96
80-27-52-4	2	505.6	849.4	51.0	1596.7	10	1	5	6.9	−8.5	1.2	−0.2	2548.8	2	1.2	1	100
80-27-75-4	1	504.6	832.3	83.7	1579.2	11	1	7	5.8	−7.3	0.7	−0.6	1316.5	2	1.1	1	91
80-27-74-4	1	491.6	812.9	68.4	1544.0	11	2	5	6.3	−7.6	1.0	−0.5	1809.6	2	0.8	1	100
80-32-52-6	2	497.7	872.5	44.6	1667.0	11	1	5	7.2	−8.5	1.4	−0.4	2997.6	2	2.2	1	100
102-31-52-6	4	552.7	974.8	82.6	1802.6	10	2	5	7.7	−10.5	1.7	−0.8	1278.1	5	3.5	1	100
102-31-51-6	3	538.7	896.4	73.1	1719.6	10	2	4	7.7	−9.4	1.7	−0.6	1529.1	5	3.1	1	100
102-31-51-4	3	524.6	885.8	79.7	1678.8	9	2	4	7.4	−9.3	1.6	−0.6	1316.1	5	1.3	1	100
80-27-54-4	2	477.5	807.4	69.1	1500.8	10	1	4	6.7	−8.1	1.2	−0.4	1689.2	1	1.0	1	100
91-27-54-4	1	473.5	773.4	70.3	1458.6	9	1	5.7	5.5	−6.9	0.8	−0.4	1750.9	1	2.4	1	100
92-27-54-4	2	485.6	806.4	72.4	1532.1	9	1	4	6.7	−8.0	1.3	−0.5	1520.6	1	1.1	1	100
94-27-54-4	2	503.6	788.3	60.2	1536.9	8	1	4	6.9	−8.1	1.4	−0.2	2167.3	1	1.5	1	100
95-27-54-4	1	471.6	751.9	66.5	1453.0	8	1	4	6.2	−7.2	1.2	−0.3	1792.3	1	5.5	1	100
Veklury	5	600.6	894.9	260.9	1721.0	16 *	4	17.9	1.0	−4.7	−0.9	−3.2 *	33.3	6		1	34
Lagevrio	0	329.3	579.5	253.6	998.7	7	4	13.3	−1.5	−2.1	−1.1	−2.3	39.0	4		2	47
Nirmatrelvir	0	499.5	713.4	183.4	1423.3	8	2.3	11.3	0.1	−2.6	−1.3	−1.3	36.4	4		2	56
Ritonavir	11	720.9	1110.9 *	134.3	2177.9 *	18 *	3.3	11.0	6.6 *	−8.4 *	0.8	−2.1	309.3	9 *		1	71
Dexamethasone	0	392.5	605.6	177.8	1141.4	5	3	8.2	1.9	−3.6	0.0	−1.3	204.1	4		3	80
Baricitinib	0	371.4	617.9	191.1	1103.5	4	1	8.5	1.6	−4.8	−0.2	−1.5	152.7	2		3	75
Lopinavir	5	628.8	1018.2 *	121.2	1992.2	16 *	4	9.5	5.8	−6.9 *	0.6	−1.8	339.0	8		1	80

^a^ designed IPCL analogues and known antiviral agents, Table 6; ^b^ drug likeness, number of property descriptors (24 out of the full list of 49 descriptors of QikProp, ver. 3.7, release 14) that fall outside of the range of values for 95% of known drugs; ^c^ molecular weight in [g·mol^−1^] (range for 95% of drugs: 130–725 g.mol^−1^) [61]; ^d^ total solvent-accessible molecular surface, in [Å^2^] (probe radius 1.4 Å) (range for 95% of drugs: 300–1000 Å^2^); ^e^ hydrophobic portion of the solvent-accessible molecular surface, in [Å^2^] (probe radius 1.4 Å) (range for 95% of drugs: 0–750 Å^2^); ^f^ total volume of molecule enclosed by solvent-accessible molecular surface, in [Å^3^] (probe radius 1.4 Å) (range for 95% of drugs: 500–2000 Å^3^); ^g^ number of non-trivial (not CX3), non-hindered (not alkene, amide, small ring) rotatable bonds (range for 95% of drugs: 0–15); ^h^ estimated number of hydrogen bonds that would be donated by the solute to water molecules in an aqueous solution. Values are averages taken over several configurations, so they can assume non-integer values (range for 95% of drugs: 0.0–6.0); ^I^ estimated number of hydrogen bonds that would be accepted by the solute from water molecules in an aqueous solution. Values are averages taken over several configurations, so they can assume non-integer values (range for 95% of drugs: 2.0–20.0); ^j^ logarithm of partitioning coefficient between n-octanol and water phases (range for 95% of drugs: −2–6.5); ^k^ logarithm of predicted aqueous solubility, logS. S in [mol·dm^−3^] is the concentration of the solute in a saturated solution that is in equilibrium with the crystalline solid (range for 95% of drugs: −6.0–0.5); ^l^ logarithm of predicted binding constant to human serum albumin (range for 95% of drugs: −1.5–1.5); ^m^ logarithm of predicted brain/blood partition coefficient (range for 95% of drugs: −3.0–1.2); ^n^ predicted apparent Caco-2 cell membrane permeability in Boehringer-Ingelheim scale in [nm s^−1^] (range for 95% of drugs: < 25 poor, > 500 nm s^−1^ great); ^o^ number of likely metabolic reactions (range for 95% of drugs: 1–8); ^p^ predicted inhibition constants ${IC}_{50}^{pre}$. The ${IC}_{50}^{pre}$ was predicted from computed ΔΔ*G*_com_ using the regression equation B shown in Table 3; ^q^ human oral absorption (1–low, 2—medium, 3—high); ^r^ percentage of human oral absorption in gastrointestinal tract (<25%—poor, >80% high); (*) star indicating that the property descriptor value falls outside the range of values for 95% of known drugs.

## Data Availability

The original contributions presented in this study are included in the article/Supplementary Material. Further inquiries can be directed to the corresponding author.

## Supplementary Materials

The following supporting information can be downloaded at: https://www.mdpi.com/article/10.3390/life16010006/s1.

## Abbreviations

The following abbreviations are used in this manuscript:

2D	Two-dimensional
3CL^pro^	3-Chymotrypsin-like Protease
3D	Three-dimensional
ACE2	Angiotensin Converting Enzyme II
ADME	Absorption, distribution, metabolism, and excretion
Ar	Ring aromatic
CAMD	Computer-aided molecular design
CFFII	Class II consistent force field
COVID-19	Coronavirus disease 2019
ΔE_int_	Interaction energy
GFE	Gibbs free energy
ΔΔG_com_	Relative GFE of formation of enzyme–inhibitor complex E:I*
ΔΔG_sol_	Solvation component of the relative GFE
HBA	Hydrogen bond acceptor
HBD	Hydrogen bond donor
ΔΔH_MM_	Enthalpy component of the relative GFE
HOA	Human oral absorption
HYD	Hydrophobic
HYD-Al	Hydrophobic aliphatic
${IC}_{50}^{exp}$	Observed half-maximal inhibitory concentration
${IC}_{50}^{pre}$	Predicted half-maximal inhibitory concentration
IPCL**_x_**	Inhibitors included in TS and in VS
MD	Molecular dynamics
MERS	Middle East respiratory syndrome
MM	Molecular mechanics
MM-PBSA	Molecular mechanics–Poisson–Boltzmann surface area
PDB	Protein Data Bank
PH4	Pharmacophore model
QSAR	Quantitative structure–activity relationship
RMSD	Root mean square deviation
RNA	Ribonucleic acid
SARS	Severe acute respiratory syndrome
SARS-CoV-2	Severe acute respiratory syndrome coronavirus 2
TS	Training set
∆∆TS_vib_	Vibrational entropy contribution of the relative GFE
VS	Validation set
