# Supplementary material for: In Silico Optimization of Inhibitors of the 3-Chymotrypsin-like Protease of SARS-CoV-2
Source: Life (Basel). 2025 Dec 19;16(1):6. doi: 10.3390/life16010006 (PMC12843385; doi:10.3390/life16010006)
Supplement: Supplementary file 1 [file life-16-00006-s001.zip › life-4015056-supplementary.pdf]

Research Article, LIFE - Basel

Supplementary Data

In Silico Optimization of Inhibitors of the 3-Chymotrypsin-like Protease of SARS-CoV-2

Issouf Fofana [a], Brice Dali [a,b], Mawa Koné [c], Katarina Sujova [d], Eugene Megnassan [a,b,c,e,f], Stanislav Miertus [e,g,]* and Vladimir Frecer [d,]*

[a] Laboratoire de Physique Fondamentale et Appliquée (LPFA), University of Abobo Adjamé (now Nangui Abrogoua), Abidjan, Côte d'Ivoire; fofetude@yahoo.fr, megnase@yahoo.com

[b] Laboratoire des Sciences de la Matière de l'Environnement et de l'énergie Solaire (LASMES), UFR SSMT Université Félix Houphouët Boigny, Abidjan, Côte d'Ivoire; dalibrice@yahoo.fr

[c] Laboratoire de Constitution et de Réaction de la Matière (LCRM), UFR SSMT Université Félix Houphouët Boigny, 22 BP 582 Abidjan 22 ; kone_m2001@yahoo.fr

[d] Department of Physical Chemistry of Drugs, Faculty of Pharmacy, Comenius University Bratislava, SK-83232 Bratislava, Slovakia; sujova23@uniba.sk; frecer@fpharm.uniba.sk

[e] International Centre for Applied Research and Sustainable Technology, Bratislava SK-84104, Slovakia;

[f] International Centre for Theoretical Physics, ICTP-UNESCO, Strada Costiera, Trieste, Italy;

[g] Department of Biotechnologies, Faculty of Natural Sciences, University of SS. Cyril and Methodius, SK-91701 Trnava, Slovakia; stanislav.miertus@ucm.sk

AUTHOR INFORMATION

FOFANA Issouf, ORCID: 0009-0004-9000-9224

DALI Brice, ORCID: 0009-0007-7326-8971

KONÉ Mawa, ORCID: 0009-0002-5771-1789

SUJOVA Katarina, ORCID: 0009-0002-2915-7485

MEGNASSAN Eugene, ORCID: 0000-0003-1505-5277

MIERTUS Stanislav, ORCID: 0000-0001-6766-4571

FRECER Vladimir, ORCID: 0000-0001-9378-5860

34

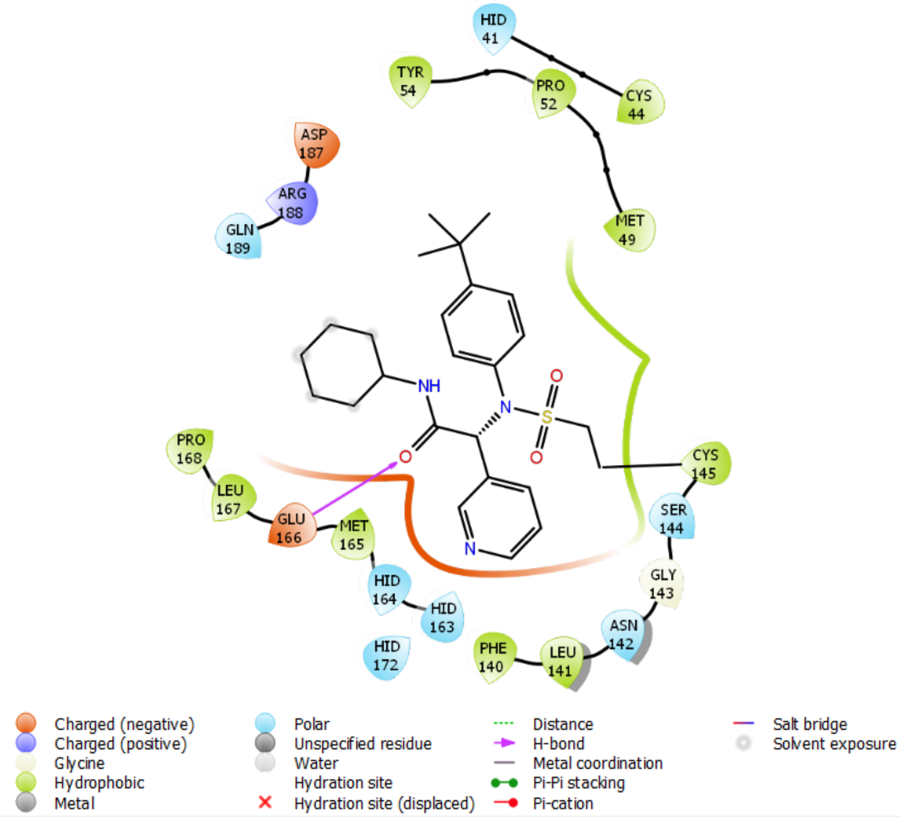
HID 41
TYR 54
PRO 52
CYS 44
ASP 187
ARG 188
GLN 189
MET 49
NH
N
S
O
O
O
N
PRO 168
LEU 167
GLU 166
MET 165
CYS 145
SER 144
GLY 143
HID 164
HID 163
HID 172
PHE 140
LEU 141
ASN 142
Charged (negative)
Charged (positive)
Glycine
Hydrophobic
Metal
Polar
Unspecified residue
Water
Hydration site
Hydration site (displaced)
Distance
H-bond
Metal coordination
Pi-Pi stacking
Pi-cation
Salt bridge
Solvent exposure

A

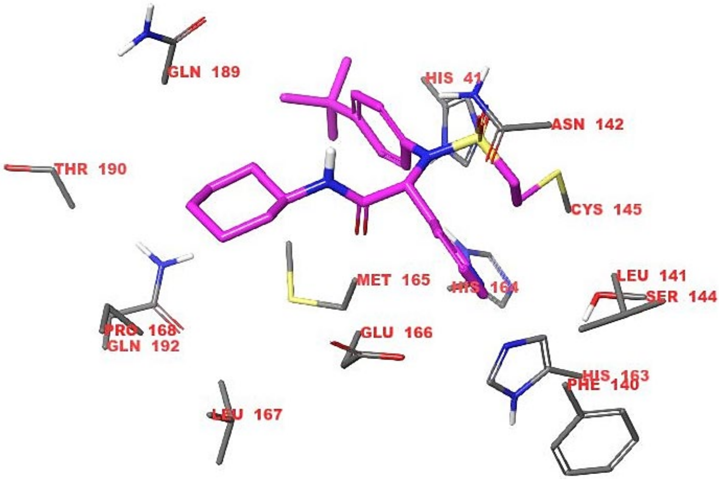
GLN 189
HIS 41
ASN 142
THR 190
CYS 145
MET 165
HIS 164
LEU 141
SER 144
PRO 168
GLN 192
GLU 166
HIS 163
PHE 140
LEU 167

B

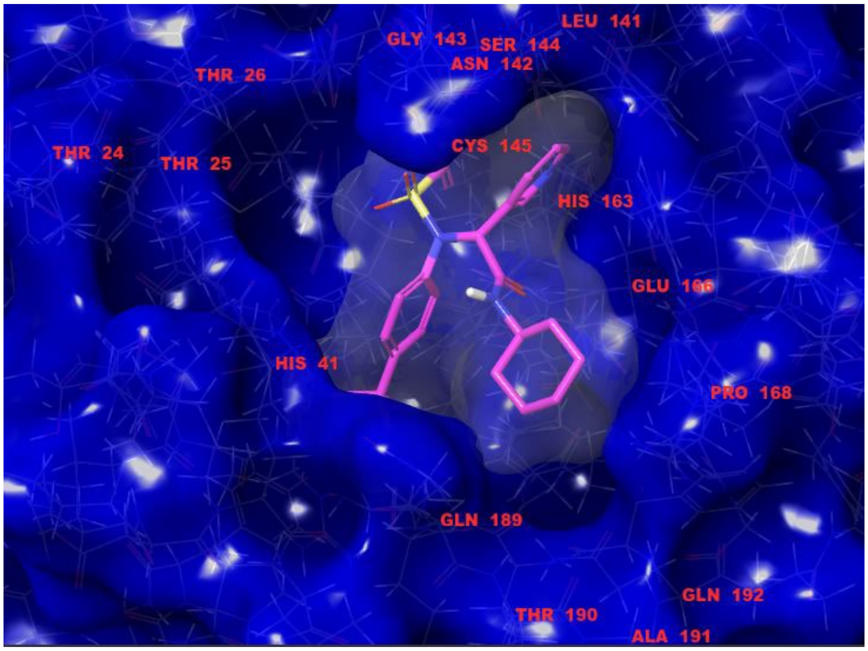

C

Figure S1. **A.** 2D schematic interaction diagram of inhibitor **14a** (IPCL6) [1] at the active site of the 3CL^{pro} protease of SARS-CoV-2. **B.** 3D structure of the 3CL^{pro} active site with bound inhibitor IPCL6. **C.** Solid molecular surface of 3CL^{pro} (in blue) with bound inhibitor IPCL6. The molecular surface of the inhibitor IPCL6 (light grey) defines the volume occupied at the active site of 3CL^{pro}. Carbon atoms of the ligand IPCL6 are coloured magenta. The side chains of the interacting residues and the ligand are represented as sticks.

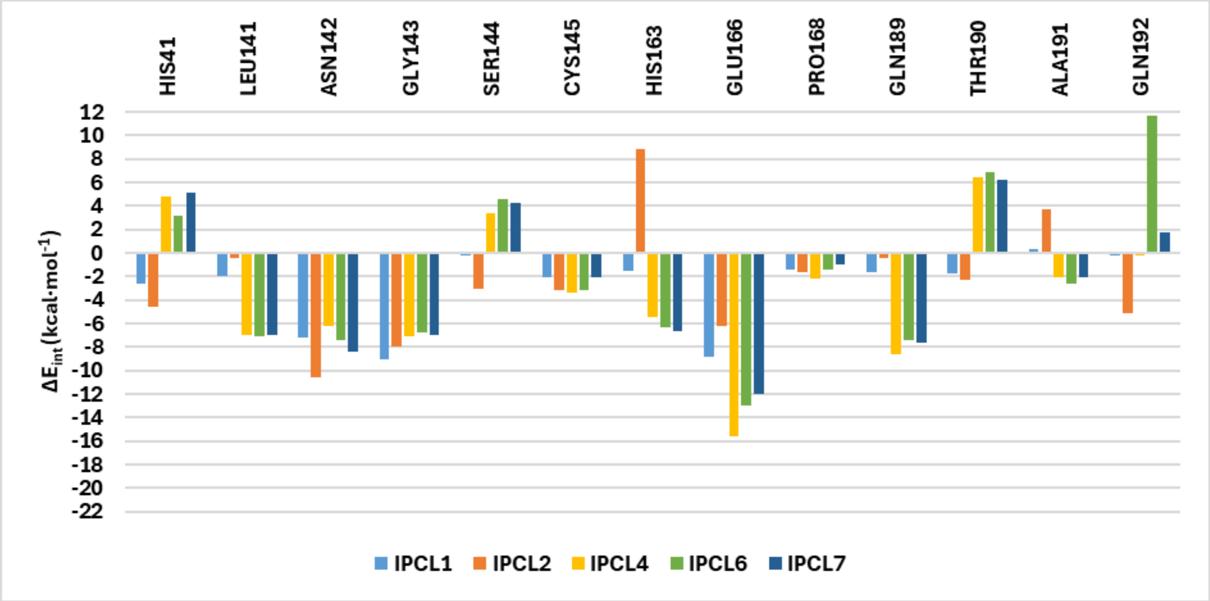

A

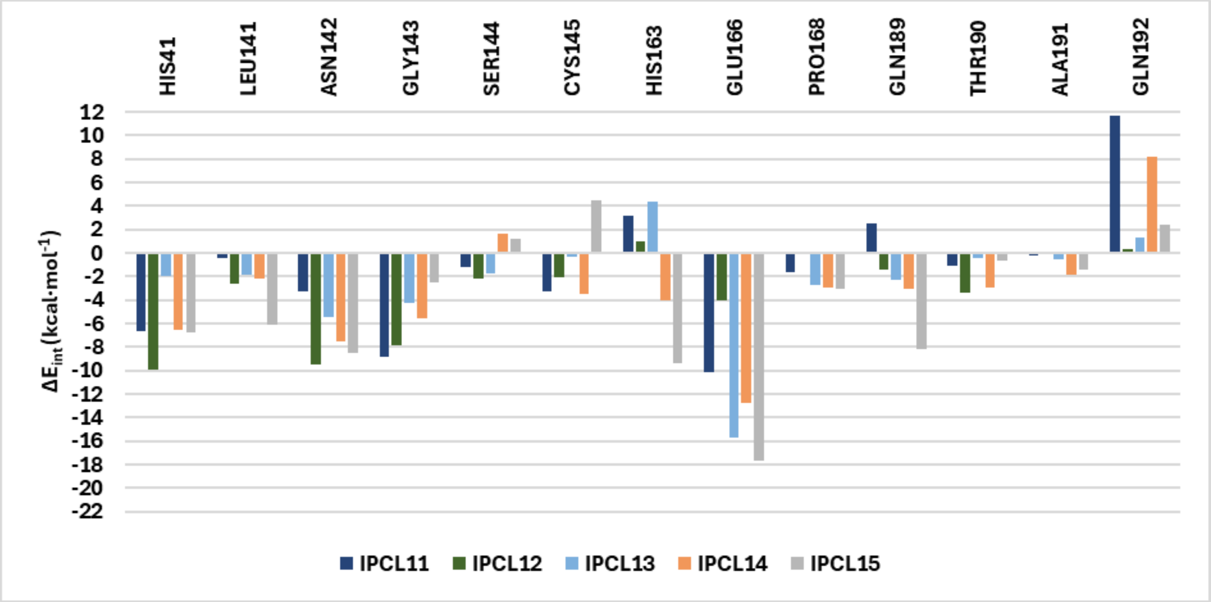

B

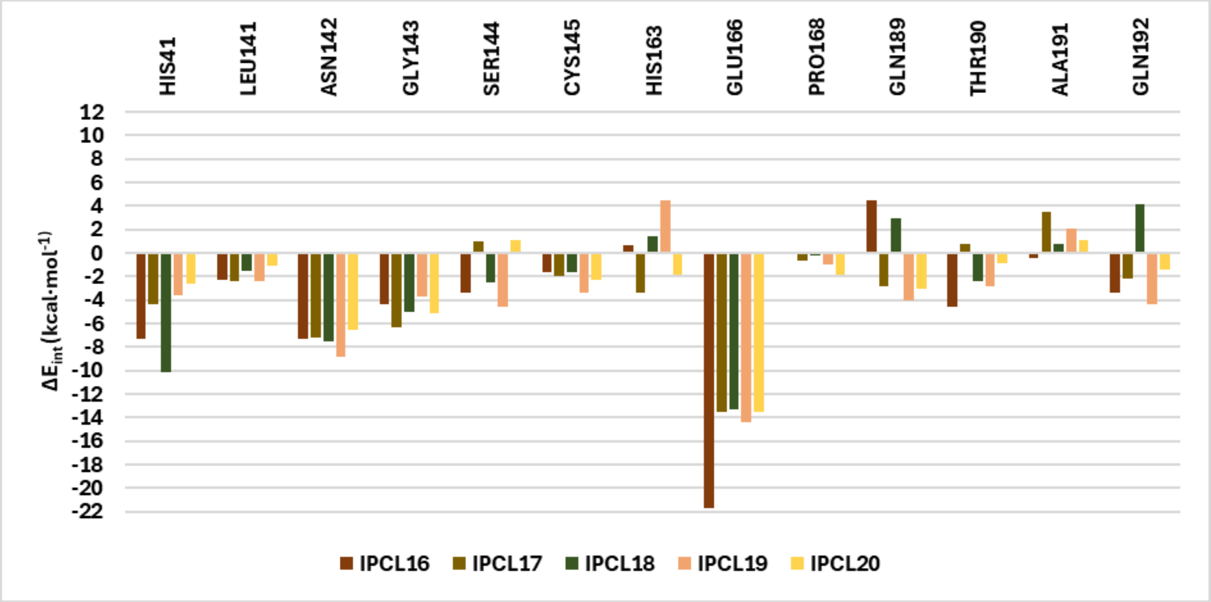

C

Figure S2. Molecular mechanics enzyme-inhibitor interaction energy ΔE_{int} contributions (in kcal·mol^{-1}) are shown for selected active site residues of 3CL^{pro} and selected training set IPCL analogues [1]. **A.** The most active IPCL inhibitors. **B.** Moderately active inhibitors. **C.** Less active inhibitors. The colour coding refers to the IPCL inhibitors, as indicated in the legend.

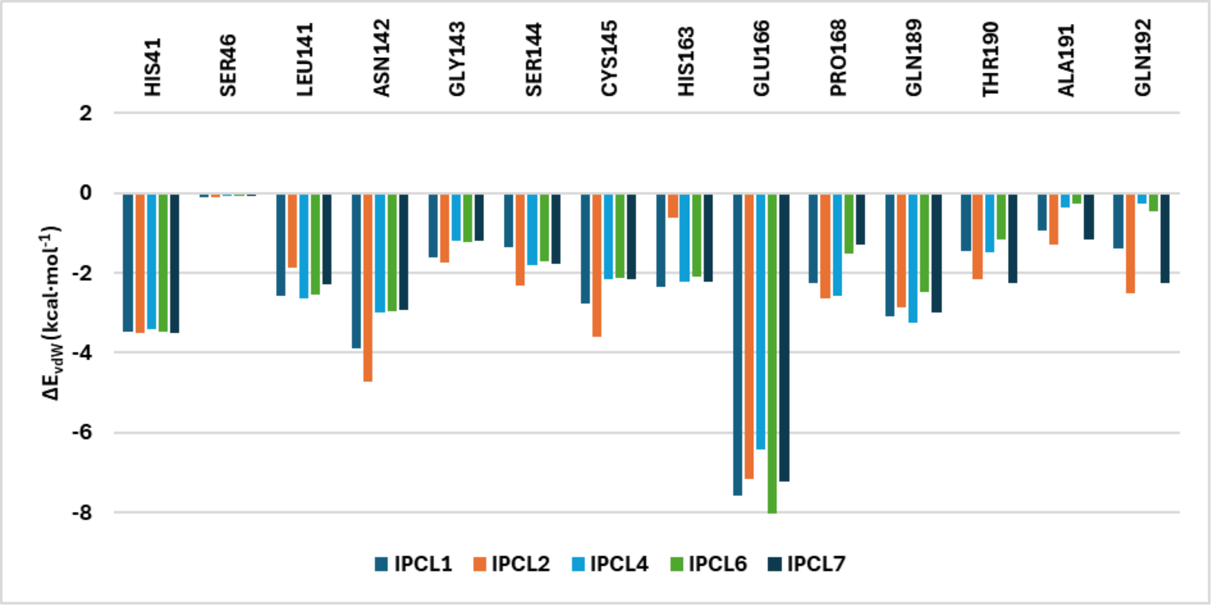

HIS41
SER46
LEU141
ASN142
GLY143
SER144
CYS145
HIS163
GLU166
PRO168
GLN189
THR190
ALA191
GLN192
2
0
-2
-4
-6
-8
ΔE_{vdW} (kcal·mol^{-1})
IPCL1
IPCL2
IPCL4
IPCL6
IPCL7

A

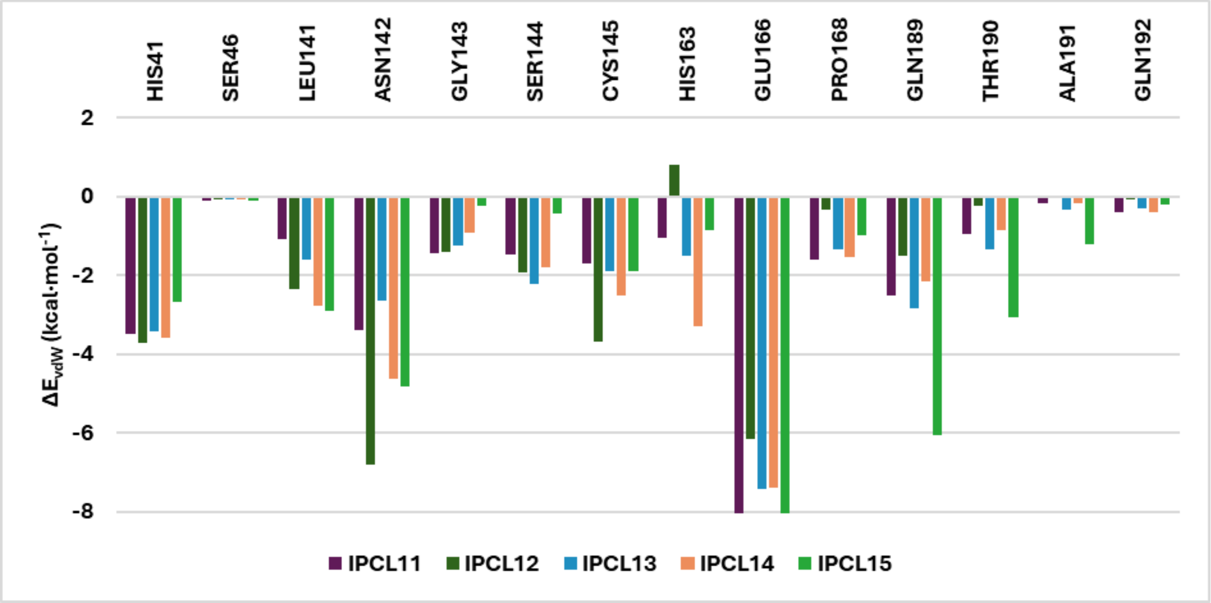

HIS41
SER46
LEU141
ASN142
GLY143
SER144
CYS145
HIS163
GLU166
PRO168
GLN189
THR190
ALA191
GLN192
2
0
-2
-4
-6
-8
ΔE_{vdW} (kcal·mol^{-1})
IPCL11
IPCL12
IPCL13
IPCL14
IPCL15

B

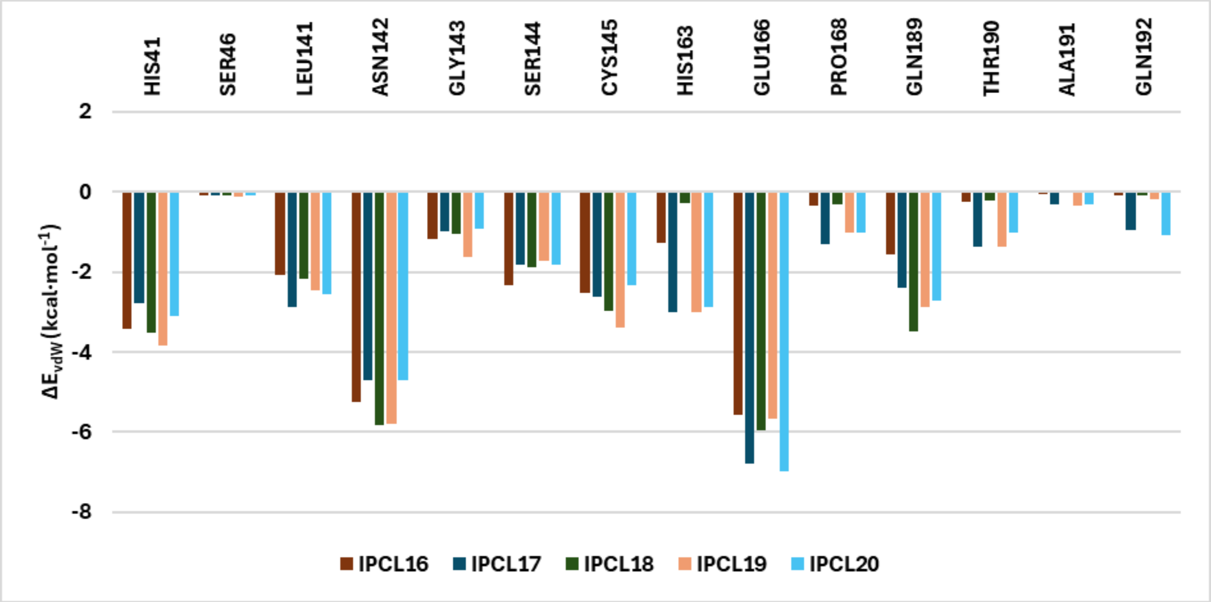

C

Figure S3. The van der Waals contributions to the enzyme-inhibitor interaction energy (ΔE_{vdW}, in kcal·mol^{-1}) are shown for selected active site residues of 3CL^{pro} and selected training set IPCL analogues [1]. **A.** The most active IPCL inhibitors. **B.** Moderately active inhibitors. **C.** Less active inhibitors. The colour coding refers to the IPCLs, as indicated in the legend.

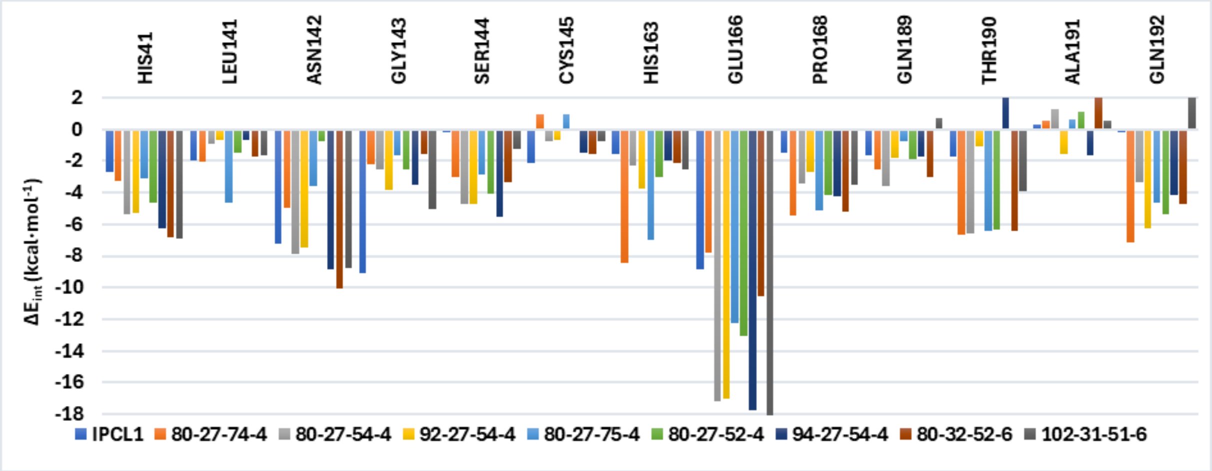

A

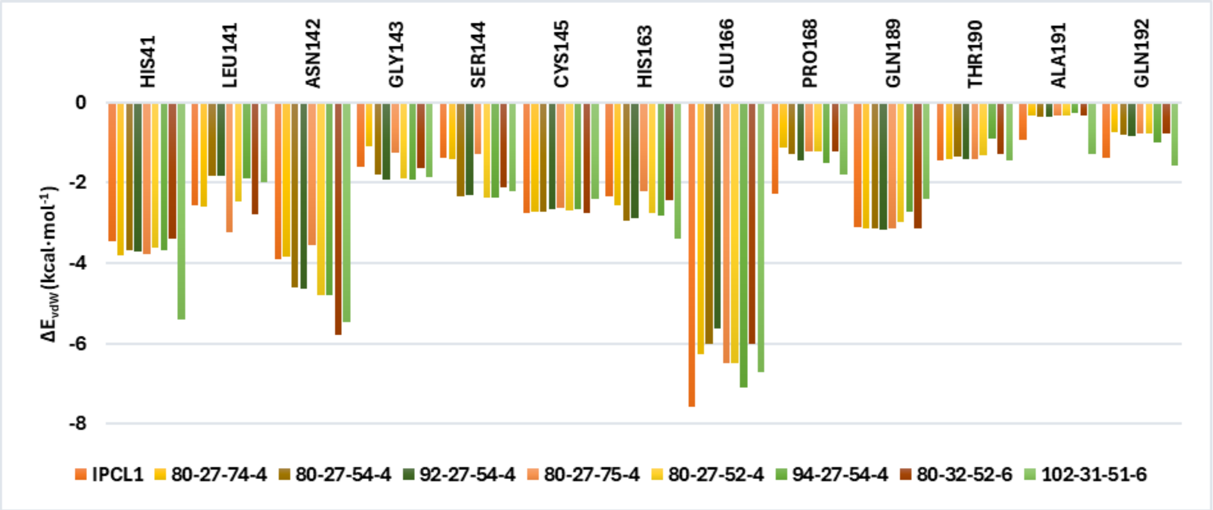

B

Figure S4. A. Molecular mechanics enzyme-inhibitor interaction energy ΔE_{int} contributions (in kcal·mol^{-1}) are shown for selected active site residues of 3CL^{pro} and best designed new IPCL analogues. **B.** The van der Waals component of the intermolecular interaction energy ΔE_{vdW} is given for selected active site residue contributions. Results are presented for IPCL1 and eight new IPCL analogues (the colour coding refers to the inhibitors, as indicated in the legend).

Table S1. Predicted ADME-related properties of the 39 IPCL virtual hits and known antiviral agents either in clinical use or currently undergoing clinical testing computed by QikProp [2].

IPCL$_x$ [a]	#stars [b]	M_w [c] [g.mol^{-1}]	S_{mol} [d] [Å^2]	$S_{mol,hfo}$ [e] [Å^2]	V_{mol} [f] [Å^3]	RotB [g]	HB_{don} [h]	HB_{acc} [i]	$logP_{o/w}$ [j]	$logS_{wat}$ [k]	$logK_{HSA}$ [l]	logB/B [m]	BIP_{caco} [n] [nm.s^{-1}]	#meta [o]	IC_{50}^{pre} [nM]	HOA [q]	%HOA [r]
IPCL1	1	512.1	848.6	98.3	1568.7	10	1	8.5	4.9	-6.7	0.4	-1.0	899.3	3	170*	1	96
79-32-57-22	0	492.7	837.3	144.7	1626.7	13	4.0	8.7	3.4	-4.6	0.0	-1.6	192.4	4	20.3	3	88
85-32-57-22	0	528.7	880.2	145.6	1684.5	13	4.0	8.7	3.9	-5.6	0.1	-1.6	190.4	4	13.5	2	78
85-30-57-22	0	563.1	847.4	153.3	1678.3	13	4.0	8.7	4.1	-5.4	0.1	-1.4	164.8	4	23.7	2	78
87-32-57-22	0	510.7	838.4	144.6	1641.3	13	4.0	8.7	3.6	-4.8	0.0	-1.5	193.6	4	17.1	2	76
79-31-57-22	0	510.7	883.0	144.4	1676.1	13	4.0	8.7	3.8	-5.5	0.1	-1.7	193.7	4	14.4	2	77
86-32-57-22	0	527.1	848.0	144.5	1654.3	13	4.0	8.7	3.7	-5.1	0.0	-1.5	195.0	4	13	2	77
86-31-57-22	0	545.1	857.3	143.7	1667.3	13	4.0	8.7	4.0	-5.4	0.1	-1.4	198.3	4	103.6	2	78
109-31-57-22	0	563.1	857.9	143.5	1674.0	13	4.0	8.7	4.1	-5.5	0.1	-1.4	201.3	4	177.4	2	79
88-50-57-22	0	498.6	811.7	150.2	1572.8	12	4.0	10.4	2.5	-4.1	-0.4	-1.5	174.3	5	23.3	2	82
79-32-57-4	1	510.7	904.6	121.8	1714.3	11	3.0	6.5	5.9	-7.4	1.1	-1.3	367.2	3	7.5	1	81
79-31-57-4	1	528.7	867.4	102.2	1682.0	11	3.0	6.5	6.0	-7.6	1.0	-0.9	815.8	3	9	1	88
79-48-52-4	1	488.5	802.9	67.5	1501.3	9	2.0	5.0	6.1	-7.8	0.9	-0.3	1874.6	4	11.9	1	100
111-47-73-22	0	499.6	790.1	141.7	1488.8	11	2.0	10.4	2.4	-4.2	-0.5	-1.3	220.8	8	28.3	2	83
80-27-53-22	0	490.6	814.2	95.1	1537.8	12	2.0	7.2	4.4	-5.5	0.2	-0.9	556.8	4	25	3	100
80-27-53-4	3	508.6	846.6	51.6	1578.0	10	1.0	5.0	6.9	-8.5	1.2	-0.2	2521.6	3	8	1	100
80-27-52-22	0	487.6	826.5	101.0	1566.0	12	2.0	7.2	4.5	-5.6	0.3	-1.0	481.5	3	12	3	100
80-27-52-4	2	505.6	849.4	51.0	1596.7	10	1.0	5.0	6.9	-8.5	1.2	-0.2	2548.8	2	1.2	1	100
80-27-75-4	1	504.6	832.3	83.7	1579.2	11	1.0	7.0	5.8	-7.3	0.7	-0.6	1316.5	2	1.1	1	91
80-27-74-4	1	491.6	812.9	68.4	1544.0	11	2.0	5.0	6.3	-7.6	1.0	-0.5	1809.6	2	0.8	1	100
77-50-76-4	0	489.6	766.6	114.2	1507.1	10	2.5	6.0	5.1	-6.2	0.7	-0.9	642.0	3	3250.6	1	94
80-32-52-6	2	497.7	872.5	44.6	1667.0	11	1.0	5.0	7.2	-8.5	1.4	-0.4	2997.6	2	2.2	1	100
78-26-58-11	0	493.6	792.6	81.9	1492.5	10	1.0	8.0	4.5	-6.1	0.3	-0.7	1353.2	2	192.2	3	100

IPCL$_x$ [a]	#stars[b]	M_w [c] [g.mol^{-1}]	S_{mol} [d] [Å^2]	$S_{mol,hfo}$ [e] [Å^2]	V_{mol} [f] [Å^3]	RotB [g]	HB_{don} [h]	HB_{acc} [i]	$logP_{o/w}$ [j]	$logS_{wat}$ [k]	$logK_{HSA}$ [l]	logB/B [m]	BIP_{caco} [n] [nm.s^{-1}]	#meta [o]	IC_{50}^{pre} [nM]	HOA [q]	%HOA [r]
102-31-52-6	4	552.7	974.8	82.6	1802.6	10	2.0	5.0	7.7	-10.5	1.7	-0.8	1278.1	5	3.5	1	100
102-31-51-6	3	538.7	896.4	73.1	1719.6	10	2.0	4.0	7.7	-9.4	1.7	-0.6	1529.1	5	3.1	1	100
102-31-51-4	3	524.6	885.8	79.7	1678.8	9	2.0	4.0	7.4	-9.3	1.6	-0.6	1316.1	5	1.3	1	100
102-31-51-1	2	492.6	847.5	124.7	1579.7	9	3.0	4.0	6.6	-8.2	1.1	-1.2	125.8	5	11.5	1	90
88-29-58-1	0	504.0	793.6	113.3	1537.8	9	2.0	7.7	5.2	-6.4	0.4	-0.8	163.0	4	10.4	1	71
103-29-58-4	1	526.0	831.3	72.2	1589.2	8	1.0	6.5	6.1	-7.8	1.0	-0.3	1536.8	6	191.1	1	94
103-29-58-1	1	494.0	787.1	109.2	1502.1	8	2.0	6.5	5.6	-6.8	0.6	-0.8	181.2	6	833.4	1	87
108-43-58-1	1	496.5	766.7	135.8	1428.8	6	3.0	6.0	5.8	-7.3	0.8	-0.9	129.2	6	967.9	1	86
98-45-63-1	2	526.4	786.9	156.4	1436.3	7	2.0	8.0	4.8	-7.0	0.1	-1.0	67.1	6	6024.8	1	75
97-45-63-1	1	494.4	759.6	158.5	1375.8	7	2.0	8.0	4.3	-6.5	0.0	-1.1	63.7	5	2653.6	1	85
80-27-54-4	2	477.5	807.4	69.1	1500.8	10	1.0	4.0	6.7	-8.1	1.2	-0.4	1689.2	1	1	1	100
91-27-54-4	1	473.5	773.4	70.3	1458.6	9	1.0	5.7	5.5	-6.9	0.8	-0.4	1750.9	1	2.4	1	100
92-27-54-4	2	485.6	806.4	72.4	1532.1	9	1.0	4.0	6.7	-8.0	1.3	-0.5	1520.6	1	1.1	1	100
94-27-54-4	2	503.6	788.3	60.2	1536.9	8	1.0	4.0	6.9	-8.1	1.4	-0.2	2167.3	1	1.5	1	100
95-27-54-4	1	471.6	751.9	66.5	1453.0	8	1.0	4.0	6.2	-7.2	1.2	-0.3	1792.3	1	5.5	1	100
101-27-54-4	2	511.6	819.1	63.0	1553.4	10	1.0	4.0	7.2	-8.4	1.3	-0.3	2093.5	2	104	1	100
107-27-54-4	2	502.5	805.9	87.1	1489.2	9	2.0	4.0	6.4	-8.0	1.1	-0.6	1123.7	3	38	1	93
Veklury	5	600.6	894.9	260.9	1721.0	16*	4	17.9	1.0	-4.7	-0.9	-3.2*	33.3	6		1	34
Lagevrio	0	329.3	579.5	253.6	998.7	7	4	13.3	-1.5	-2.1	-1.1	-2.3	39.0	4		2	47
Nirmatrelvir	0	499.5	713.4	183.4	1423.3	8	2.3	11.3	0.1	-2.6	-1.3	-1.3	36.4	4		2	56
Ritonavir	11	720.9	1110.9*	134.3	2177.9*	18*	3.3	11.0	6.6*	-8.4*	0.8	-2.1	309.3	9*		1	71
Dexamethasone	0	392.5	605.6	177.8	1141.4	5	3	8.2	1.9	-3.6	0.0	-1.3	204.1	4		3	80
Baricitinib	0	371.4	617.9	191.1	1103.5	4	1	8.5	1.6	-4.8	-0.2	-1.5	152.7	2		3	75
Lopinavir	5	628.8	1018.2*	121.2	1992.2	16*	4	9.5	5.8	-6.9*	0.6	-1.8	339.0	8		1	80

[a] designed IPCL analogues, Tables 6;
[b] drug likeness. Number of property descriptors (24 out of the full list of 49 descriptors of QikProp. ver. 3.7. release 14) that fall outside of the range of values for 95% of known drugs;
[c] molecular mass in [g.mol^{-1}] (range for 95% of drugs: 130 - 725 g.mol^{-1}) [2];
[d] total solvent-accessible molecular surface. in [Å^2] (probe radius 1.4 Å) (range for 95% of drugs: 300 - 1000 Å^2);
[e] hydrophobic portion of the solvent-accessible molecular surface. in [Å^2] (probe radius 1.4 Å) (range for 95% of drugs: 0 - 750 Å^2);
[f] total volume of molecule enclosed by solvent-accessible molecular surface. in [Å^3] (probe radius 1.4 Å) (range for 95% of drugs: 500 - 2000 Å^3);
[g] number of non-trivial (not CX3). non-hindered (not alkene. amide. small ring) rotatable bonds (range for 95% of drugs: 0 - 15);
[h] estimated number of hydrogen bonds that would be donated by the solute to water molecules in an aqueous solution. Values are averages taken over several configurations. so they can assume non-integer values (range for 95% of drugs: 0.0 - 6.0);
[i] estimated number of hydrogen bonds that would be accepted by the solute from water molecules in an aqueous solution. Values are averages taken over several configurations. so they can assume non-integer values (range for 95% of drugs: 2.0 - 20.0);
[j] logarithm of partitioning coefficient between n-octanol and water phases (range for 95% of drugs: -2 - 6.5);
[k] logarithm of predicted aqueous solubility logS. S in [mol·dm^{-3}] is the concentration of the solute in a saturated solution that is in equilibrium with the crystalline solid (range for 95% of drugs: -6.0 - 0.5);
[l] logarithm of predicted binding constant to human serum albumin (range for 95% of drugs: -1.5 - 1.5);
[m] logarithm of predicted brain/blood partition coefficient (range for 95% of drugs: -3.0 - 1.2);
[n] predicted apparent Caco-2 cell membrane permeability in Boehringer-Ingelheim scale in [nm s^{-1}] (range for 95% of drugs: < 25 poor. > 500 nm s^{-1} great);
[o] number of likely metabolic reactions (range for 95% of drugs: 1 - 8);
[p] predicted inhibition constants IC_{50}^{pre}. The IC_{50}^{pre} was predicted from computed $\Delta\Delta G_{com}$ using the regression equation B shown in Table 3;
[q] human oral absorption (1 - low. 2 - medium. 3 - high);
[r] percentage of human oral absorption in gastrointestinal tract (<25% - poor. >80% high);
(*) star indicating that the property descriptor value falls outside the range of values for 95% of known drugs.

Table S2. Ensemble averages of the total and potential energy of complexes 3CL^{pro}–IPCL$_x$ for the IPCL1 [1] and best designed new analogues over 200 ns long MD simulation at 300 K.

IPCL	Chemical structure	IC_{50}^{pre} [a] [nM]
**IPCL1**	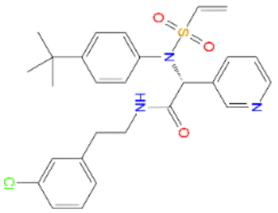	170 *
**80-27-74-4**	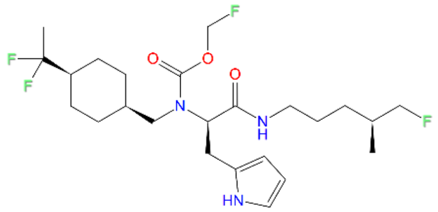	0.8
**80-27-54-4**	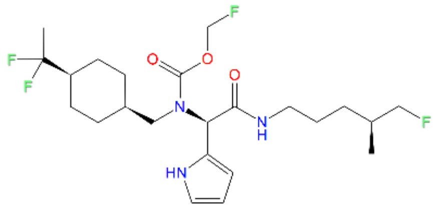	1.0
**80-27-75-4**	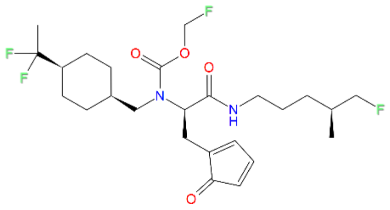	1.1
**92-27-54-4**	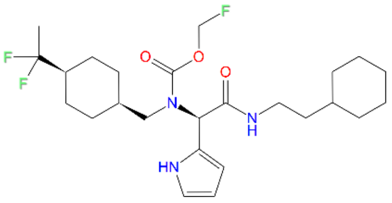	1.1
**80-27-52-4**	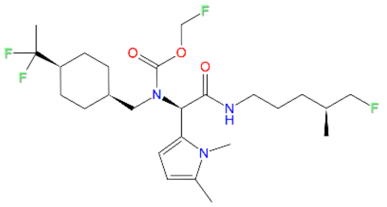	1.2

IPCL	Chemical structure	IC_{50}^{pre} [a] [nM]
**102-31-51-4**	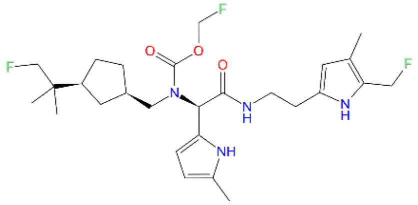	1.3
**94-27-54-4**	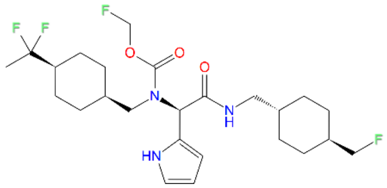	1.5
**80-32-52-6**	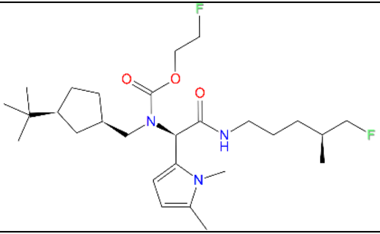	2.2
**91-27-54-4**	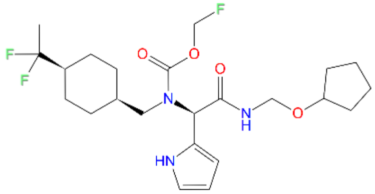	2.4
**102-31-51-6**	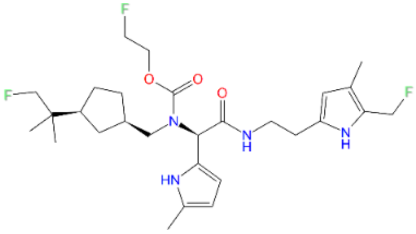	3.1
**102-31-52-6**	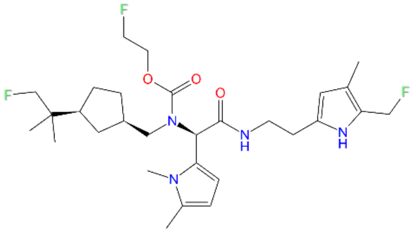	3.5
**95-27-54-4**	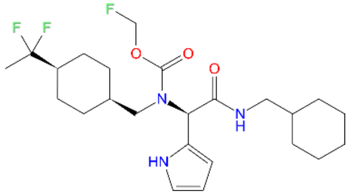	5.5

[a] IC_{50}^{pre} were predicted by the QSAR model (Table 3, Eq. B) for the new analogues. * Experimental IC_{50}^{exp} is given for the IPCL1 [1].

IPCL1 **80-27-74-4**

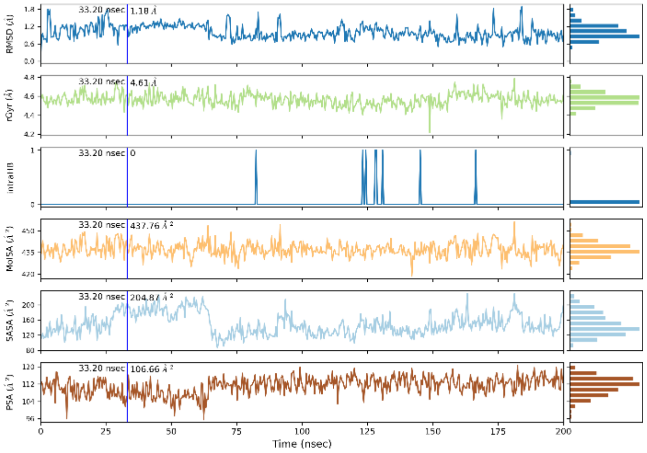

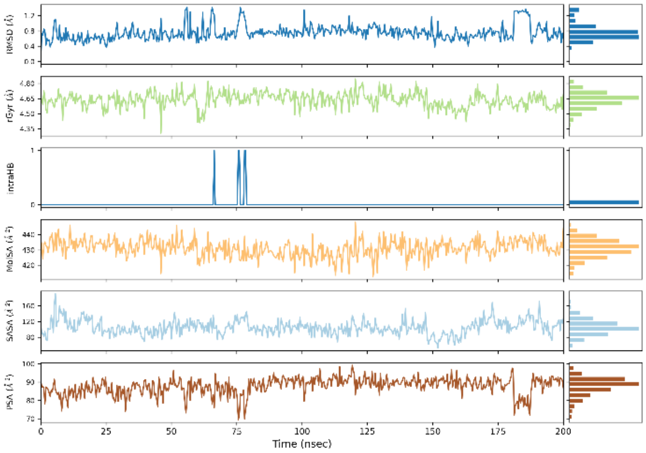

80-27-54-4 **80-27-75-4**

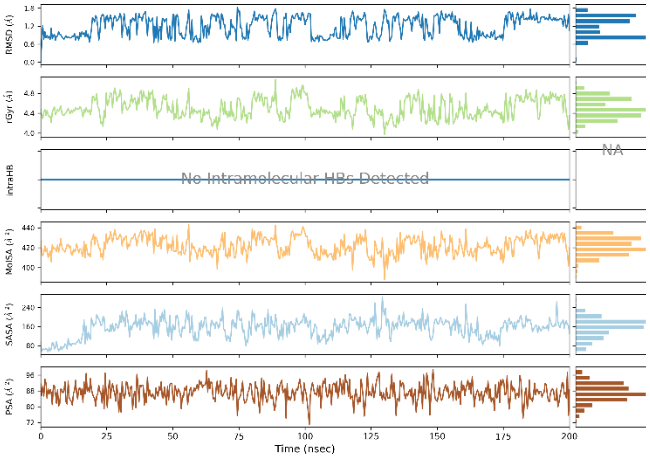

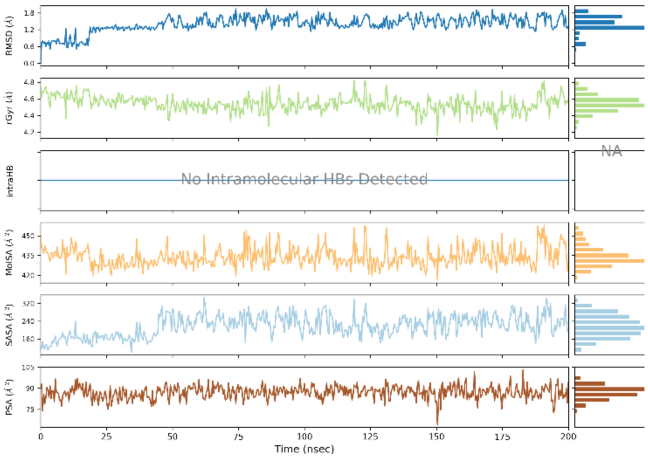

92-27-54-4 **80-27-52-4**

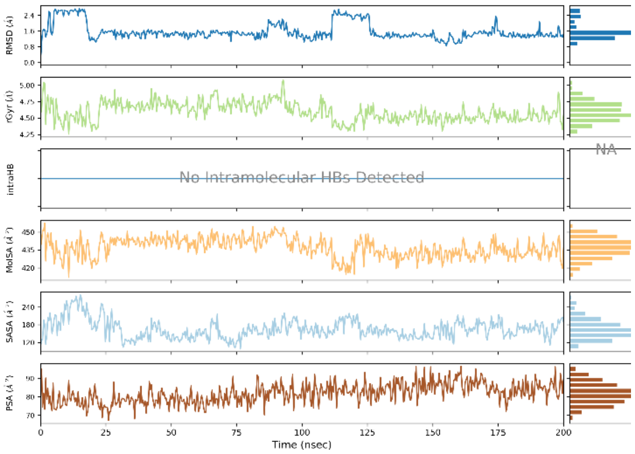

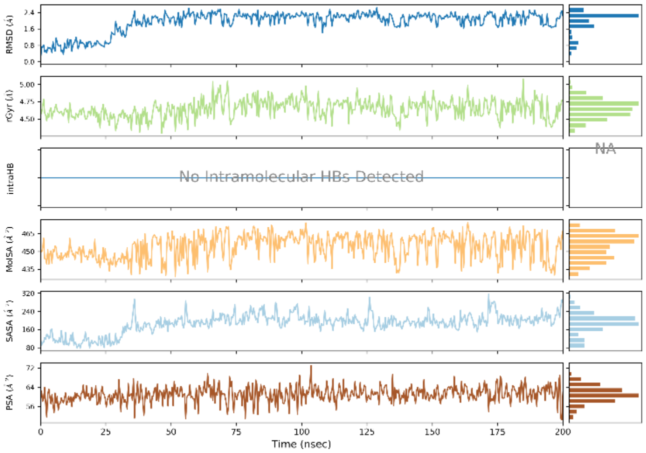

102-31-51-4 **94-27-54-4**

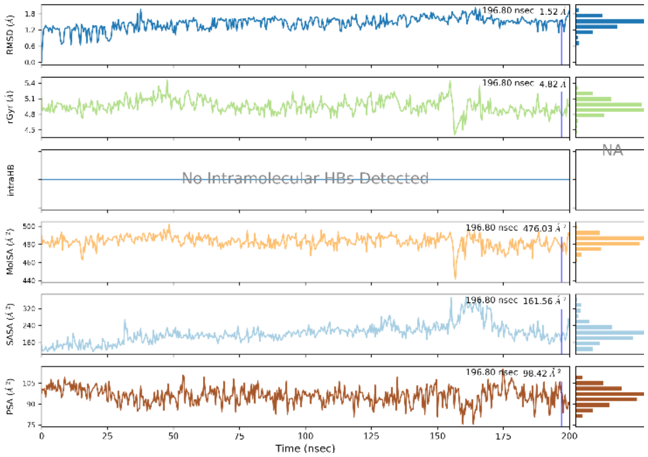

80-32-52-6

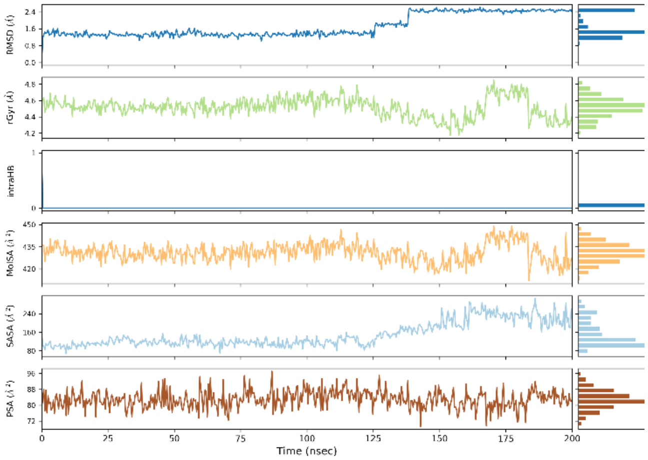

91-27-54-4

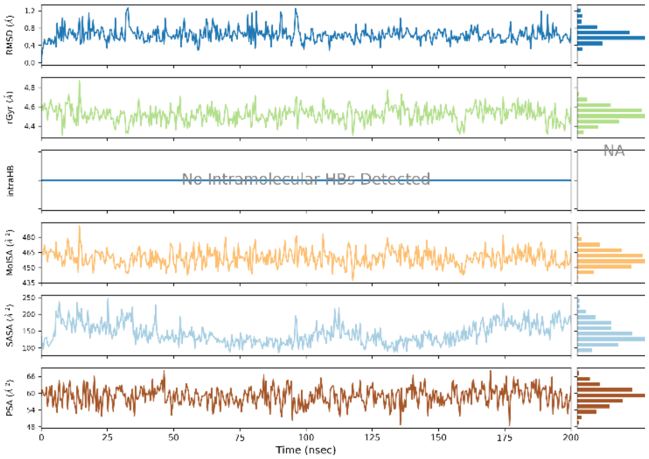

102-31-51-6

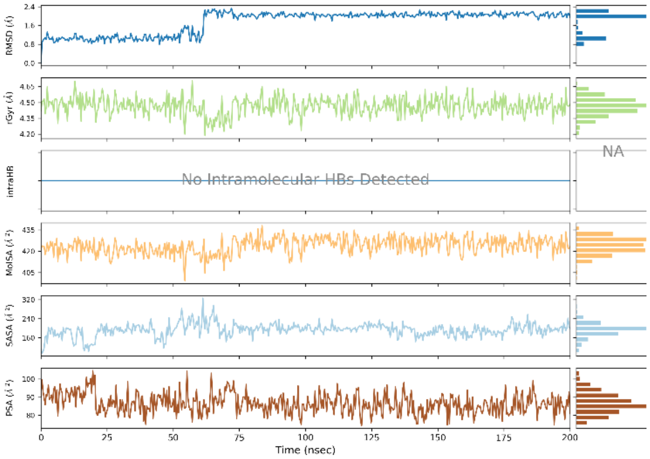

102-31-52-6

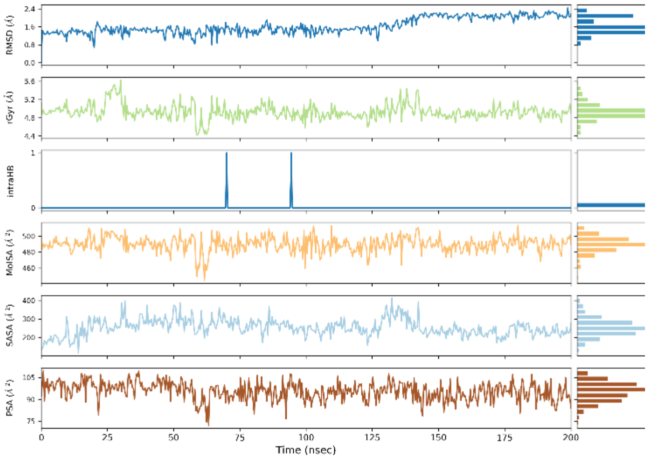

95-27-54-4

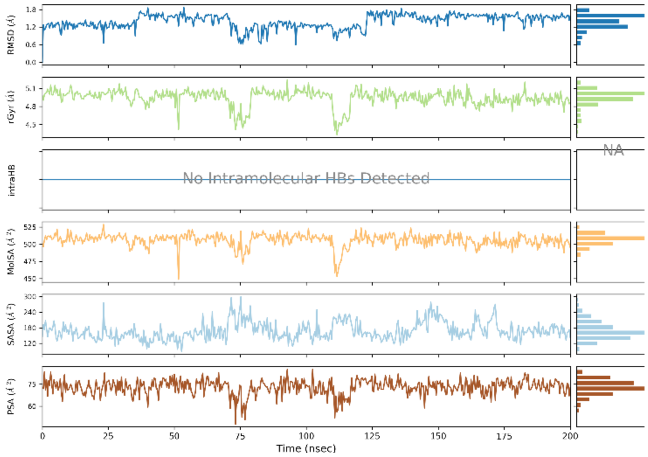

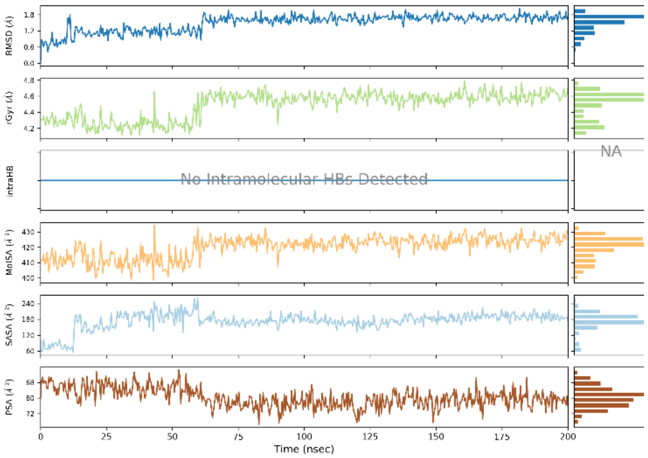

Figure S5. Molecular properties of IPCL1 and 12 IPCLs (Table S2) bound to SARS-CoV-2 3CL^{pro} were monitored over 200 ns of MD simulation time. For each inhibitor, root mean square deviation (RMSD) with respect to the initial conformation of the IPCL, radius of gyration (rGyr), number of intramolecular hydrogen bonds (intraHB), molecular surface area (molSA), solvent-accessible surface area (SASA), and polar surface area (PSA), were plotted (top to bottom in each diagram).

IPCL1

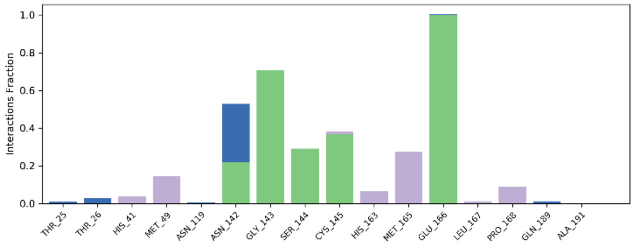

80-27-74-4

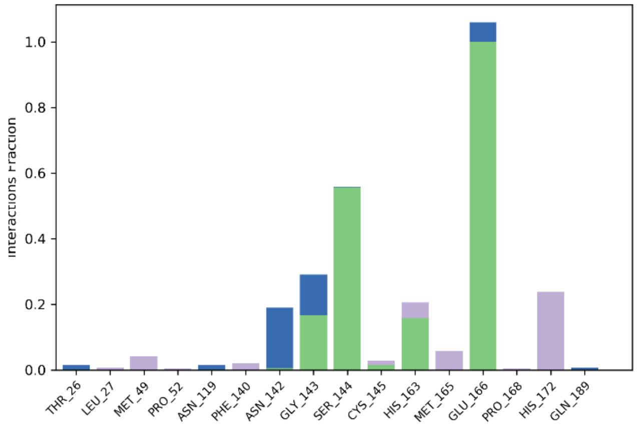

80-27-54-4

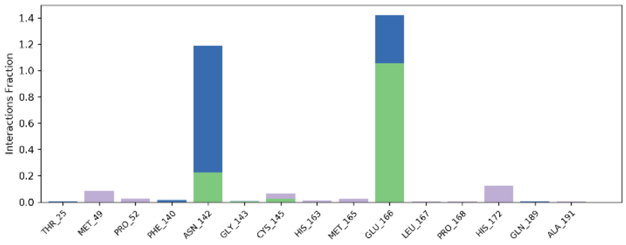

80-27-75-4

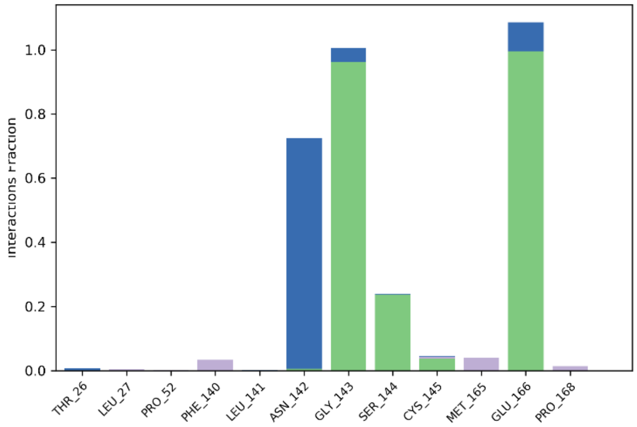

92-27-54-4 **80-27-52-4**

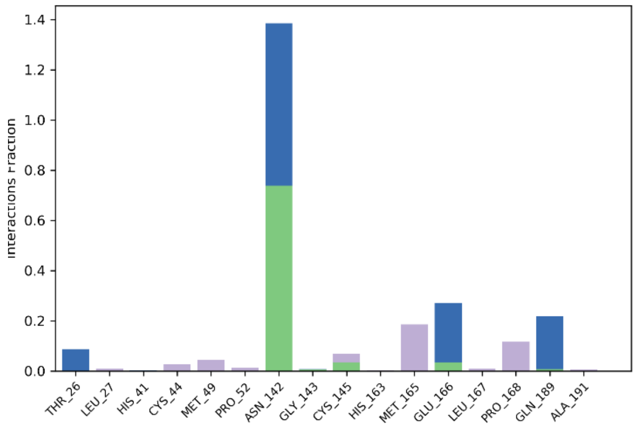

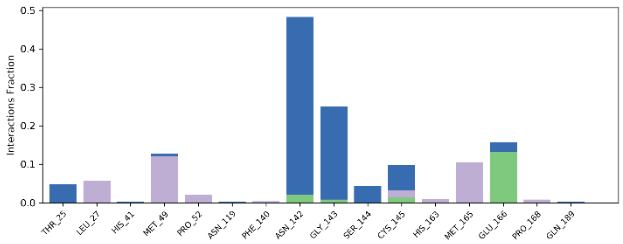

102-31-51-4 **94-27-54-4**

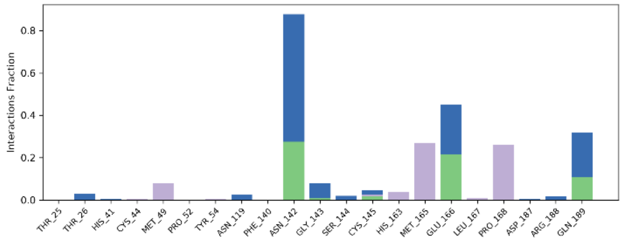

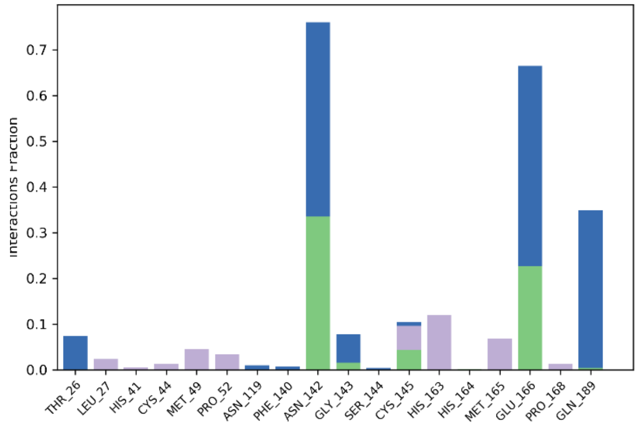

80-32-52-6 **91-27-54-4**

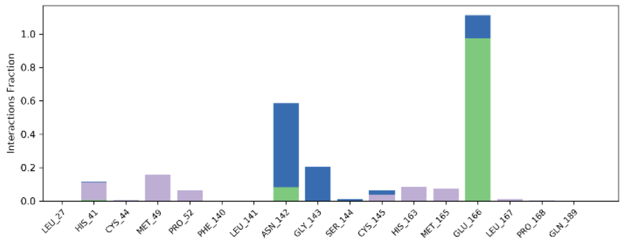

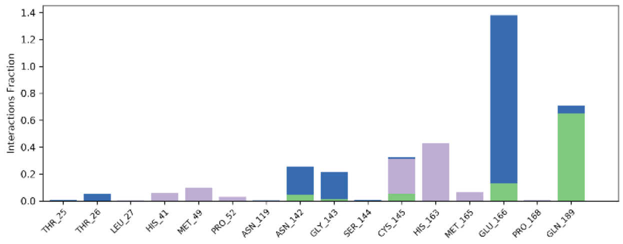

102-31-51-6 **102-31-52-6**

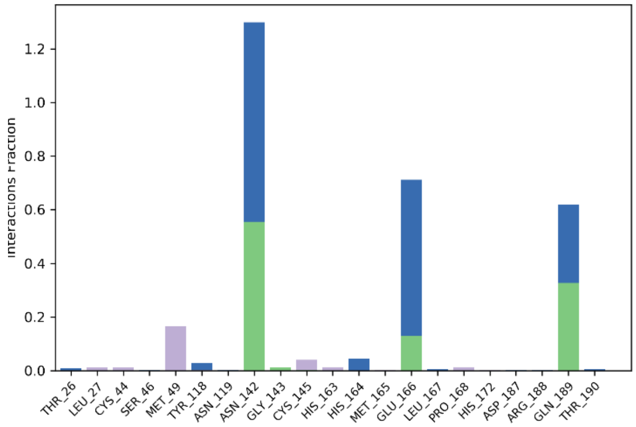

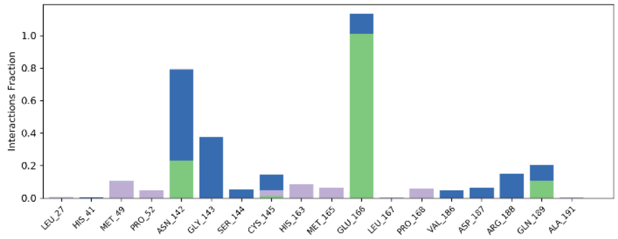

95-27-54-4

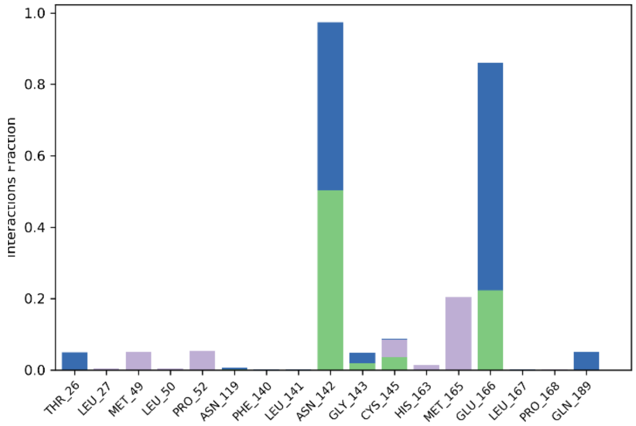

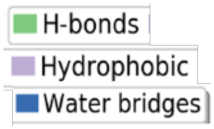

Figure S6. Contributions of individual active site residues to the $3CL^{pro}$-$IPCL_x$ binding energy present during at least during 20% of the MD simulation time.

IPCL1 **80-27-74-4**

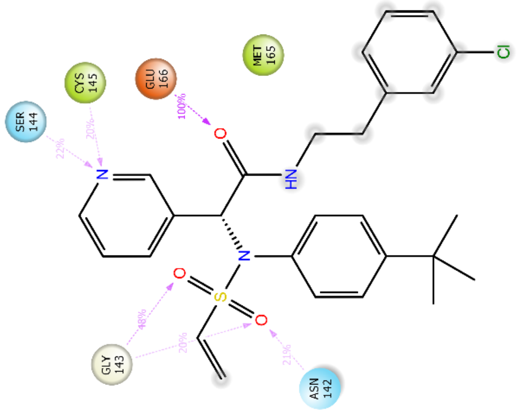

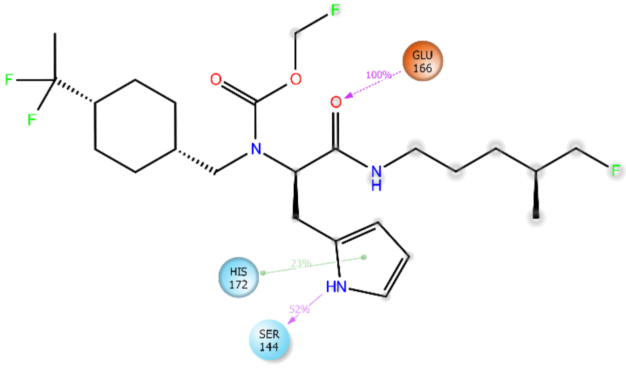

80-27-54-4 **80-27-75-4**

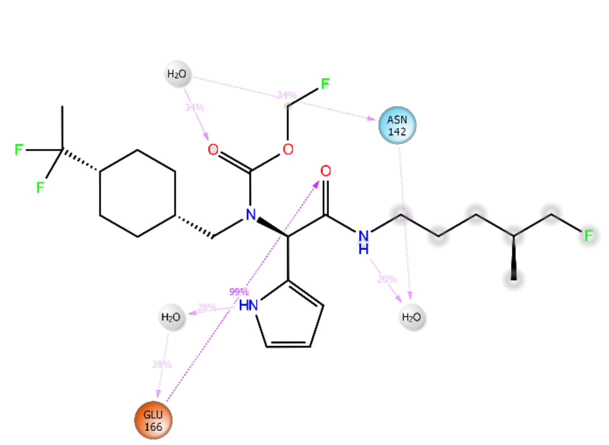

92-27-54-4

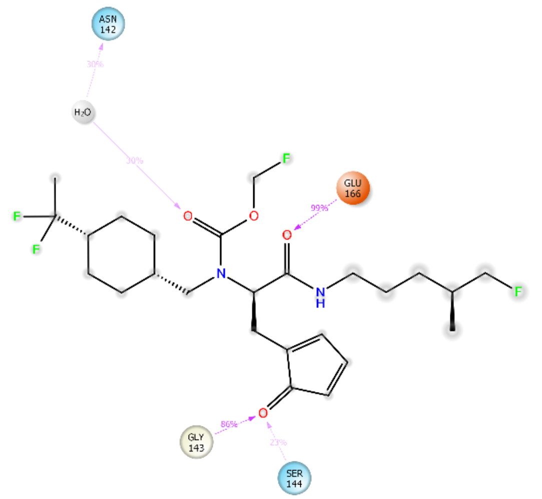

80-27-52-4

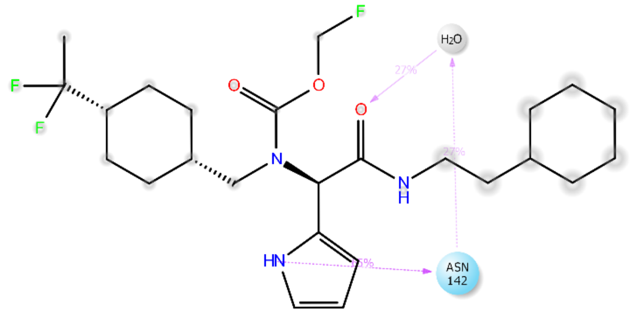

102-31-51-4

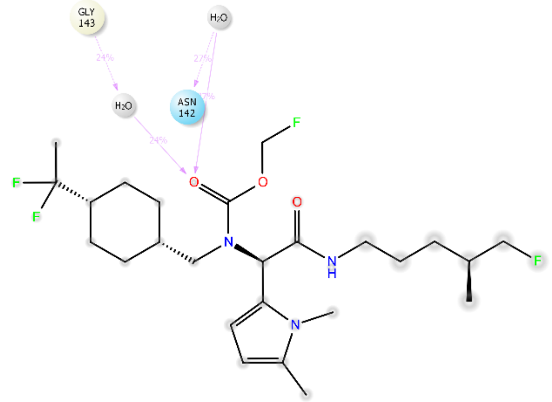

94-27-54-4

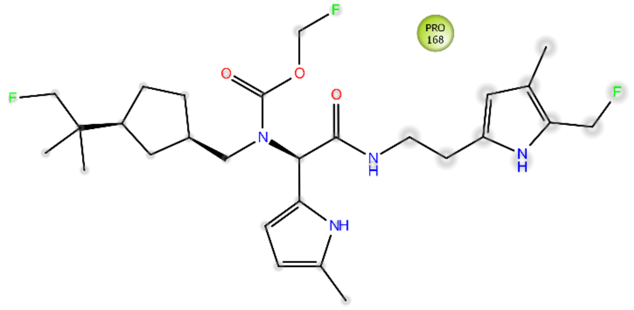

80-32-52-6

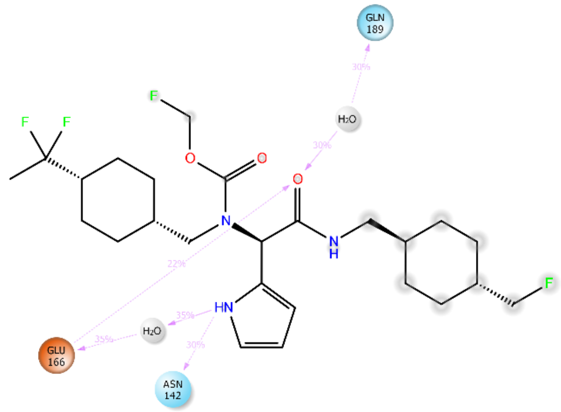

91-27-54-4

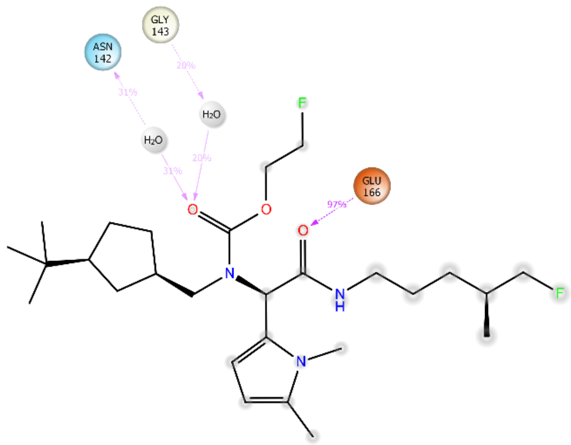

102-31-51-6

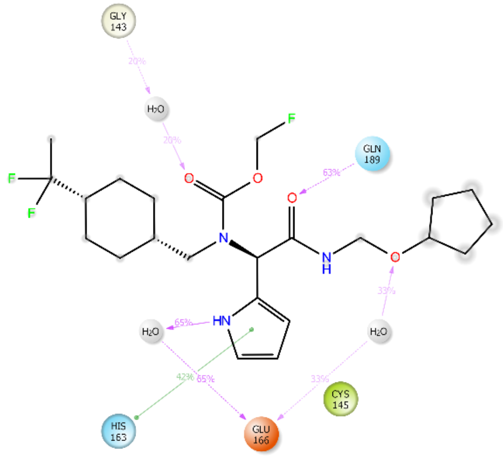

102-31-52-6

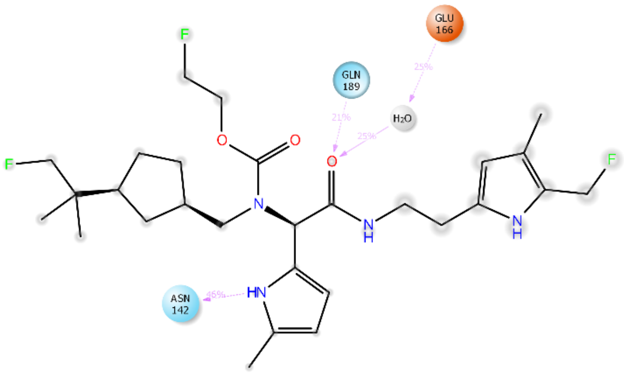

95-27-54-4

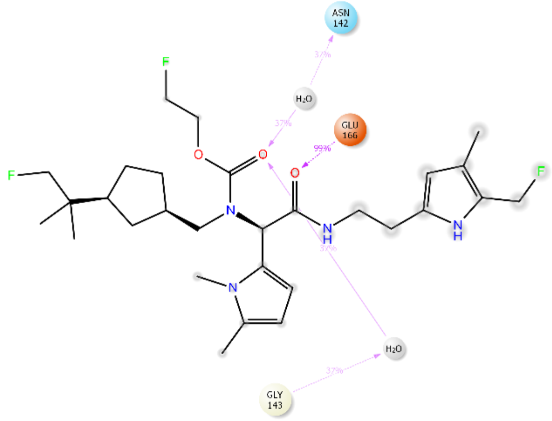

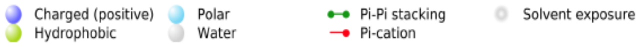

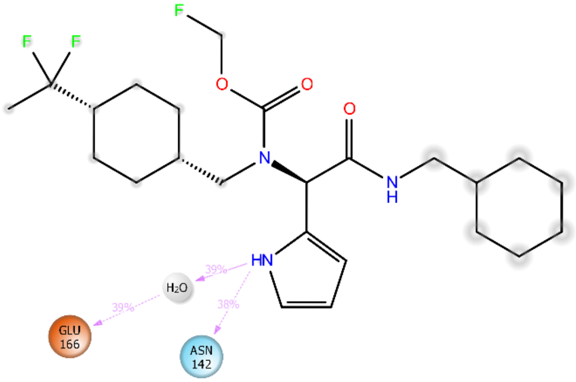

Figure S7. The 2D schemes present attractive interactions prevalent between the functional groups of the 12 inhibitor candidates and the individual residues of the active site of SARS-CoV-2 3CL[pro]. These interactions were observed in at least 20 % of the analysed 500 frames of the MD trajectory (i.e. in 40 ns).

REFERENCES

1. J.K. Stille, J. Tjutrins, G. Wang *et al.* **2022**. Design, synthesis and in vitro evaluation of novel SARS-CoV-2 3CLpro covalent inhibitors, *Eur J Med Chem.* 229, art. No. 114046 (doi: 10.1016/j.ejmech.2021.114046)

2. QikProp, 6.5 (Release 139); Schrödinger LLC: New York, NY, USA, **2019**.
